# Implementation effectiveness, barriers, and real-world outcomes of neuromuscular training programs for ACL injury prevention in female athletes: systematic review with narrative synthesis using SWiM framework

**DOI:** 10.3389/fpubh.2026.1743075

**Published:** 2026-02-24

**Authors:** Yongzhe Gao, Lu Qi, Tongwu Yu

**Affiliations:** 1 Woosuk University, Wanju-gun, Republic of Korea; 2 Guilin University of Electronic Technology, Guilin, China; 3 Anhui Communications Vocational and Technical College, Hefei, China

**Keywords:** ACL injury prevention, female athletes, implementation science, neuromuscular training, real-world effectiveness, synthesis without meta-analysis, systematic review

## Abstract

**Background:**

ACL injuries disproportionately affect female athletes. Neuromuscular training (NMT) is effective in controlled studies, but real-world adoption and adherence remain poor.

**Objective:**

To synthesize evidence on implementation effectiveness, barriers/facilitators, and real-world outcomes of NMT for ACL prevention in female athletes using narrative synthesis.

**Methods:**

Following SWiM, we searched PubMed, SPORTDiscus, Scopus, and Web of Science (2014–2025) for controlled studies reporting implementation outcomes. NMT was defined as a multi-component intervention including ≥2 of: plyometrics, strength, balance/proprioception, agility, or sport-specific movement training. Barriers/facilitators were thematically analyzed using CFIR, and findings were organized within the RE-AIM framework. Study quality was assessed with MMAT 2018 and used for sensitivity and certainty appraisal rather than exclusion.

**Results:**

Thirteen studies (*n* = 2,847) were included. Implementation quality was consistently associated with program effectiveness. One high-quality study suggested that delivering NMT ≥ 2 times/week reduced ACL injury risk by 85% in amateur female soccer players (HR = 0.15, 95% CI 0.03–0.73, *p* = 0.019), though this threshold needs validation across settings. Implementation strategies showed a gradient: stakeholder-engaged models with professional support achieved higher compliance (73.5–85.6%) than education-only approaches. In one school-based study with limited professional support, coaches were an implementation bottleneck (52.5% compliance), despite high athlete compliance when programs were delivered (87.8%). Educational institutions showed higher implementation outcomes (89–100%) than community settings (52.5–85.6%). Key barriers were time constraints, competing priorities, and insufficient ongoing support; facilitators included professional supervision, organizational commitment, and systematic stakeholder engagement. ACL-specific outcomes were reported in 3 studies and broader injury outcomes in 6; compliance was measured in 12/13 studies (92%).

**Conclusion:**

Implementation quality appears to be a major determinant of real-world effectiveness for ACL prevention, potentially as important as program selection. Comprehensive support strategies outperform passive dissemination, underscoring the need to prioritize implementation science and systematic professional development to sustain injury-prevention programs.

**Systematic review registration:**

https://inplasy.com/inplasy-2025-6-0057/, identifier INPLASY202560057.

## 1 Introduction and background

Anterior cruciate ligament (ACL) injuries represent one of the most significant challenges in contemporary sports medicine, with female athletes experiencing injury rates 2 to 8 times higher than their male counterparts, predominantly through non-contact mechanisms (1–3). This difference generally appears post-pubertal and has been related to multiple anatomical and biomechanical variables, although there is a high degree of heterogeneity among populations. The proposed contributing factors are a broader pelvic structure, smaller joint dimensions, increased joint laxity, smaller ACL size, and other neuromuscular movement patterns during pivoting, jumping, and landing tasks, with the relative importance of each factor varying significantly across individuals and athletic groups (1–3). These factors create a unique vulnerability profile placing female athletes at elevated risk in sports characterized by cutting, jumping, and pivoting movements, particularly soccer, basketball, handball, and volleyball.

The magnitude of this public health challenge is substantial, with the global ACL reconstruction market estimated to grow from $7.45 billion in 2024 to $10.48 billion by 2029 (4). Beyond financial burden, ACL injuries impose profound consequences often requiring ≥6–9 months for return to sport post-reconstruction (5, 6), psychological disturbances, social isolation, and reduced academic performance (1, 3, 7). Most concerning is the substantially elevated risk of premature osteoarthritis, particularly with concomitant meniscal damage, setting the stage for decades of reduced quality of life (5, 8). The risk of subsequent ACL injuries further compounds these concerns, highlighting the complex interplay between initial injury, surgical intervention, and long-term athletic participation.

Evidence-based neuromuscular training (NMT) programs have emerged as promising interventions for injury prevention. The scientific foundation has evolved since the 1990s from single-component approaches to comprehensive, multi-modal programs integrating diverse training elements (5, 9, 10). Contemporary NMT programs include ACL-specific interventions such as Dynamic Neuromuscular Analysis (DNA) training and Prevent Injury and Enhance Performance (PEP), as well as broader injury prevention programs like FIFA 11 and FIFA 11 + that address multiple lower-extremity injury risks while incorporating ACL prevention components. These programs systematically address ACL injury risk through structured combinations of plyometric training, strengthening exercises, balance enhancement, and movement pattern correction. The efficacy evidence is compelling, with Sugimoto et al. demonstrating relative risk reductions of 73.4% for non-contact ACL injuries and 43.8% for overall ACL injuries among female athletes participating in NMT programs (5). Efficacy studies conducting numbers-needed-to-treat (NNT) analyses indicate 108 to 120 athletes need to complete programs to prevent one ACL injury during one competitive season (11). Critically, these NNT values were obtained under controlled research conditions with high implementation fidelity; actual effectiveness in practice may vary significantly based on implementation quality, a distinction that highlights the importance of implementation-oriented research (11–13). Although these efficacy-based values might seem large, they are more favourable when considering overall benefits that go beyond ACL prevention, such as decreases in other knee and ankle injuries and possible performance gains.

Despite robust efficacy evidence under controlled conditions, a persistent implementation gap continues to plague the field. Female sports participation has reached historical highs (14, 15), yet ACL injury rates have not decreased proportionally to prevention evidence strength, indicating fundamental challenges in translating research findings into routine practice. This disconnect represents a classic implementation science challenge extending beyond program availability to encompass complex organizational, individual, and contextual factors influencing adoption, adherence, and sustainability.

Implementation challenges are multifaceted and rooted in complex sports participation realities across diverse settings. Although significant literature demonstrates the beneficial effects of neuromuscular training (1, 7, 9, 10), other studies have documented inconclusive or counterintuitive results, such as increased rates of injury in intervention groups compared to controls (3, 7, 16). Such counterintuitive results can be due to methodological constraints including insufficient sample sizes, lack of follow-up, differences in baseline injury risk, or, critically to this review, poor implementation fidelity and not necessarily inefficiency of intervention. These results highlight the need to differentiate between intervention efficacy and implementation quality in the interpretation of prevention research findings (1, 3, 7, 9). Compliance assessment has revealed significant methodological challenges, with supervision evaluation and documentation varying considerably across investigations, creating a complex landscape of implementation approaches that may significantly influence outcomes.

Heterogeneity in implementation approaches encompasses fundamental aspects of program delivery, duration, frequency, and intensity, making it challenging to identify components that maximize prophylactic effects. Different NMT types have been applied across various sports, age groups, and study designs, creating a complex matrix of variables influencing both implementation feasibility and effectiveness (17, 18). Financial and time burden associated with program implementation presents additional challenges, as administration costs and time commitments must be weighed against potential benefits.

Current systematic reviews and meta-analyses have focused almost exclusively on efficacy outcomes, determining whether NMT programs work under controlled research conditions. While essential for establishing evidence base, this research provides limited guidance for practitioners, coaches, and organizations implementing programs in real-world settings. The complexity of implementation does not only involve program adoption but also several measurable constructs: organizational readiness (measured by leadership commitment, change climate, and available resources), resource availability (including personnel time, equipment, and funding), stakeholder buy-in (measured by knowledge, attitudes, and behavioural intentions), training requirements (including initial competency development and continued skill maintenance), and long-term sustainability considerations [including institutionalization, adaptation, and continued fidelity monitoring (5, 7, 11, 16, 19)]. These constructs are consistent with well-known implementation science frameworks such as the Consolidated Framework of Implementation Research (CFIR) and the RE-AIM, which offer systematic methods to operationalize and measure implementation determinants and outcomes. These implementation science aspects have received insufficient systematic attention despite their critical importance for translating research evidence into meaningful population-level impact.

The theoretical framework for understanding implementation challenges draws from broader implementation science literature, recognizing that effective translation requires understanding complex, multi-level factors influencing adoption and sustained use (5, 16, 19). NMT program implementation occurs within dynamic systems including individual athletes, coaches, teams, organizations, and broader sporting systems with varying policies and support structures. This complexity cannot be adequately captured through traditional research approaches focusing primarily on intervention efficacy while treating implementation factors as secondary considerations.

Implementation science has emerged as a distinct field providing valuable frameworks for understanding effective intervention translation into routine practice. However, its application to sports injury prevention has been limited, creating a knowledge gap hampering efforts to maximize the population-level impact of evidence-based prevention programs. The heterogeneity of implementation research with varied contexts, populations, implementation strategies, and outcome measures requires synthesis approaches that preserve contextual complexity while enabling systematic evidence integration. By systematically synthesizing implementation evidence across diverse settings, populations, and implementation strategies, this review addresses critical knowledge gaps by: (1) determining real-world compliance and adherence rates; (2) identifying barriers and facilitators using established implementation science frameworks; (3) comparing real-world with controlled trial outcomes; and (4) evaluating implementation strategy effectiveness for maximizing program adoption, adherence, and sustainability across diverse contexts. This evidence provides practical guidance for practitioners, organizations, and policymakers working to bridge the research-practice gap in ACL injury prevention for female athletes.

## 2 Methods

### 2.1 Protocol registration

The systematic review protocol was registered with the International Platform of Registered Systematic Review and Meta-Analysis Protocols (INPLASY) (registration number: INPLASY202560057) on 14 June 2025 (20). The protocol provides detailed methodology including search strategy, eligibility criteria, outcome measures, and statistical analysis plan assessing the implementation effectiveness, barriers, facilitators, and real-world outcomes of neuromuscular training programs for ACL injury prevention in female athletes. The complete protocol is available at https://inplasy.com/inplasy-2025-6-0057/. The review adhered to PRISMA 2020 reporting guidelines (21). There were no deviations from the registered protocol.

### 2.2 Study design and reporting

This systematic review with narrative synthesis was designed to address the implementation-practice gap in neuromuscular training (NMT) programs for anterior cruciate ligament (ACL) injury prevention in female athletes. The study employed the Synthesis Without Meta-analysis (SWiM) reporting framework, which was specifically developed to guide clear reporting in reviews of interventions that use alternative synthesis methods for meta-analysis of effect estimates (22).

Narrative synthesis was selected over meta-analysis due to substantial heterogeneity observed in the implementation research evidence base, including: (1) varied implementation outcome definitions (compliance measured as percentage of prescribed sessions completed vs. fidelity assessed through program delivery quality; adherence tracked as longitudinal consistency vs. single-timepoint measurement); (2) inconsistent exposure metrics for injury outcomes (some studies reporting per athlete-exposure, others per training hours, and some providing only crude incidence without exposure adjustment); (3) diverse study designs (cluster randomized controlled trials vs. individual RCTs vs. controlled before-after studies, with varying levels of allocation concealment and follow-up periods); (4) heterogeneous implementation strategies (ranging from educational handouts to comprehensive multi-component professional support models) that precluded meaningful pooling; and (5) insufficient comparable quantitative data across studies, with implementation outcomes reported using different scales, timeframes, and operational definitions. These sources of heterogeneity would make pooled effect estimates potentially misleading, as they would mask critical contextual and methodological differences essential for understanding implementation processes.

The SWiM approach promotes transparent reporting of how studies are grouped, the standardised metric used for the synthesis, the synthesis method, how data are presented, a summary of the synthesis findings, and limitations of the synthesis (22, 23), improving reporting clarity while preserving the contextual complexity essential to understanding implementation processes in real-world settings. Methodological rigor was maintained through systematic application of implementation science frameworks (CFIR and RE-AIM), pre-specified synthesis methods, independent dual review processes, and explicit quality assessment using validated tools.

The review was grounded in implementation science theoretical frameworks, specifically the updated Consolidated Framework for Implementation Research (CFIR) (24, 25), which provides a menu of constructs arranged across five domains that can be used to systematically assess potential barriers and facilitators to implementation, and the RE-AIM framework (Reach, Effectiveness, Adoption, Implementation, Maintenance) (26, 27), which has been widely used for evaluation and planning programs, providing guidance for systematic assessment and feedback in implementation research.

### 2.3 Theoretical framework

This systematic review was anchored in two complementary implementation science frameworks that provide structured approaches for understanding and analysing the complex factors influencing implementation success. The CFIR is one of the most commonly used determinant frameworks to assess contextual factors that can be powerful forces working against implementation in the real world (24, 25, 28). This review employed the updated CFIR (version 2.0, 2022) (25, 26), which provides a menu of constructs arranged across five domains that can be used in a range of applications as a practical framework to help guide systematic assessment of potential barriers and facilitators (24, 28).

CFIR constructs were identified inductively during data extraction rather than pre-selected, allowing barriers and facilitators to emerge from the included studies’ reporting. During data extraction, barriers and facilitators described in each study were systematically coded to the most appropriate CFIR domain and construct based on construct definitions provided in the CFIR framework documentation (24, 28). This inductive approach ensured comprehensive capture of all implementation determinants reported in the literature while maintaining theoretical coherence through systematic framework mapping.

The five CFIR domains include: (1) Innovation Characteristics (formerly Intervention Characteristics), encompassing features such as program complexity, adaptability, and relative advantage; (2) Outer Setting, addressing external context including policy environment and competitive pressures; (3) Inner Setting, focusing on organizational culture, resources, and structural characteristics; (4) Individuals, examining knowledge, beliefs, and self-efficacy of implementers; and (5) Process, encompassing planning, engagement, execution, and evaluation activities.

The RE-AIM framework is one of the most frequently applied implementation frameworks that has been cited in over 2,800 publications (29). RE-AIM is widely used to plan and evaluate the implementation of healthcare and public health interventions, providing guidance for systematic assessment and feedback in implementation research (26, 27, 30). The five RE-AIM dimensions operate at multiple levels: reach and effectiveness address individual-level outcomes, while adoption, implementation, and maintenance encompass organizational and system-level factors. RE-AIM can and should include an assessment of long-term sustainment intentions and plans, with growing recognition of the importance of understanding long-term sustainment in a dynamic context.

These frameworks were selected for their complementary nature and established utility in implementation research. Used together, the two frameworks identify metrics for evaluating implementation success and modifiable factors that explain and enhance implementation outcomes (31). CFIR provides the theoretical structure for systematically categorizing barriers and facilitators, while RE-AIM offers an evaluative framework for organizing implementation outcomes across multiple dimensions and levels.

### 2.4 Search strategy

#### 2.4.1 Database selection and rationale

A comprehensive search strategy was developed to identify studies reporting implementation-related outcomes of neuromuscular training programs for ACL injury prevention in female athletes. Four major electronic databases were systematically searched from January 1, 2014, to May 31, 2025, to capture contemporary implementation research aligned with modern implementation science frameworks: PubMed (MEDLINE), SPORTDiscus via EBSCOhost, Scopus, and Web of Science Core Collection. Database selection was informed by the multidisciplinary nature of implementation research, which spans sports medicine, implementation science, and behavioural intervention literature (32). PubMed provided comprehensive coverage of biomedical and health sciences literature with robust MeSH vocabulary for precise implementation science concepts. SPORTDiscus offered specialized coverage of sports medicine and exercise science literature, ensuring the capture of sport-specific prevention programs. Scopus and Web of Science provided broad multidisciplinary coverage and citation tracking capabilities, essential for identifying implementation research across diverse academic fields.

#### 2.4.2 Search strategy development

Search strategies were developed using a systematic approach combining Medical Subject Headings (MeSH) terms and free-text keywords to maximize sensitivity while maintaining specificity for implementation research (32–34). The search strategy incorporated four main concept groups connected using Boolean operators: (1) ACL injury prevention concepts, (2) neuromuscular training interventions, (3) implementation science terminology, and (4) female athlete populations. Primary search concepts included: ACL injury prevention: [“anterior cruciate ligament”(MeSH) OR “ACL injury” OR “knee injuries/prevention and control”(MeSH) OR “Athletic Injuries/prevention and control”(MeSH)]; Neuromuscular training: [“Exercise Therapy”(MeSH) OR “neuromuscular training” OR “injury prevention program” OR “FIFA 11+” OR “PEP program” OR “KLIP” OR “Sportsmetrics”]; Implementation science: (“implementation” OR “compliance” OR “adherence” OR “adoption” OR “real world” OR “effectiveness” OR “barriers” OR “facilitators” OR “program delivery” OR “sustainability” OR “dissemination”); and Study population: [“Female”(MeSH) OR “female athlete” OR “women” OR “girls” OR “female”]. The Boolean logic combined these concepts using AND operators between concept groups and OR operators within concept groups. Search strategies were adapted for each database platform to optimize retrieval while maintaining consistency across sources (see Supplementary Appendix 1). Database-specific syntax accommodated variations in field codes, proximity operators, and truncation symbols while preserving search logic integrity.

### 2.5 Selection criteria

#### 2.5.1 Inclusion criteria

This review was restricted to controlled study designs specifically randomized controlled trials (RCTs), controlled clinical trials, crossover trials, and controlled before-after studies to enable systematic comparison of implementation approaches while maintaining methodological rigor (20, 35, 36). This design restriction was selected to balance the need for comparative evidence (implementation strategy A vs. B, or intervention vs. control) with recognition that implementation research often occurs in pragmatic settings where perfect randomization may not be feasible. Controlled designs provide a level of internal validity necessary for drawing meaningful conclusions about implementation strategy effectiveness while remaining more feasible than strict RCT-only requirements. Real-world effectiveness was captured through implementation outcomes (compliance rates, adherence measures, fidelity assessments, adoption patterns) and injury outcomes measured within these controlled trials conducted in naturalistic settings (schools, community sports clubs, elite training facilities), rather than highly controlled laboratory environments. This approach enabled assessment of program performance under real-world implementation conditions while maintaining comparison group rigor.

##### 2.5.1.1 Population

The participant population comprised female athletes of any age participating in organized sports, with studies involving coaches, trainers, or other program implementers also eligible when reporting implementation-related outcomes. This inclusive age approach recognized that ACL injury prevention is relevant across the developmental spectrum, from youth athletes through adult competitors.

##### 2.5.1.2 Intervention criteria

Intervention criteria specified neuromuscular training (NMT) programs designed for anterior cruciate ligament injury prevention or broader lower-extremity injury prevention that included ACL risk reduction as a component. Programs were required to include at least two components from the following training modalities: balance/proprioceptive exercises, plyometric exercises, strength training, agility training, or sport-specific movement training. This two-component minimum was established to distinguish comprehensive neuromuscular training interventions from single-modality programs (e.g., strength-only or balance-only training), recognizing that effective ACL prevention programs integrate multiple training elements that address the multifactorial nature of ACL injury risk (5, 7, 11, 16, 19). This criterion allowed inclusion of both ACL-specific programs (e.g., PEP, DNA training) and broader injury prevention programs (e.g., FIFA 11+) that incorporate multi-component neuromuscular training addressing ACL risk factors. Single-component interventions were excluded as they do not reflect current evidence-based approaches to neuromuscular injury prevention.

##### 2.5.1.3 Comparator groups

Comparator groups included standard or usual training practices, control groups receiving no intervention, alternative training programs, or different implementation strategies for the same intervention (e.g., educational-only vs. comprehensive support approaches).

##### 2.5.1.4 Outcomes

Critical to this implementation-focused review, studies were required to report quantifiable implementation outcomes such as compliance rates, adherence measures, or fidelity assessments, or provide qualitative implementation process data including barriers, facilitators, implementation strategies, sustainability measures, or real-world effectiveness outcomes measured in naturalistic settings. Studies reporting only biomechanical outcomes were included if they also provided quantifiable implementation metrics; biomechanical outcomes (e.g., knee abduction angles, ground reaction forces) served as secondary measures of neuromuscular adaptation and surrogate indicators of potential injury risk reduction, rather than primary real-world effectiveness indicators. Primary real-world effectiveness outcomes were defined as injury incidence (preferably exposure-adjusted rates such as injuries per 1,000 athlete-exposures or training hours) measured during the intervention period in naturalistic implementation settings.

##### 2.5.1.5 Additional eligibility criteria

Additional eligibility criteria included English language publications, peer-reviewed journal articles, and publication dates between January 1, 2014, and May 31, 2025, to capture contemporary implementation research aligned with modern implementation science frameworks. The 2014 start date was selected to correspond with the increasing application of implementation science frameworks to sports injury prevention research and to focus on contemporary NMT programs that reflect current evidence-based practice.

#### 2.5.2 Exclusion criteria

Studies were excluded if they focused solely on male athletes or mixed populations without separate female data analysis. Research involving only injured athletes or post-surgical rehabilitation populations was excluded, as were studies not reporting implementation-related outcomes or those using non-controlled study designs such as observational studies, case series, or case reports. While observational studies provide valuable real-world evidence, they were excluded due to the increased risk of selection bias and confounding when comparing implementation approaches without controlled allocation. Conference abstracts, editorials, commentaries, reviews, and studies reporting only biomechanical outcomes without implementation process data were excluded. Laboratory-based studies without real-world implementation context and investigations where implementation data could not be separated from efficacy results were also deemed ineligible.

#### 2.5.3 Selection process

The study selection process followed a systematic two-stage approach using the EPPI-Reviewer version 6 web platform to ensure transparency and reproducibility (37, 38). Retrieved citations from all database searches were initially imported into Zotero reference management software for comprehensive bibliographic organization and preliminary review. Following initial organization in Zotero, all retrieved records were uploaded to the EPPI-Reviewer version 6 web platform where automated duplicate removal was performed to eliminate redundant citations across databases. Implementation outcomes were operationally defined to guarantee uniform screening choices before title/abstract screening with a structured classification framework. The implementation outcomes were classified into three areas: (1) Quantitative implementation measures, such as compliance rates (percentage of prescribed sessions completed), adherence measures (consistency of program delivery over time), fidelity measures (degree to which programs were delivered as intended), adoption measures (proportion of eligible settings or individuals initiating program use), and reach measures (proportion of target population participating); (2) Implementation process measures, such as descriptions of delivery methods, implementer attributes, training methods, supervision models, and adaptation processes; and (3) Implementation determinants, such as barriers research had to report a minimum of one quantifiable implementation metric in domain 1, or present substantial qualitative data in domains 2 or 3 with enough detail to allow thematic analysis. To calibrate reviewer agreement, a screening decision tree was designed and pilot-tested on 20 randomly selected abstracts before full screening began.

The screening of the titles and abstracts was performed in the EPPI-Reviewer platform (37, 38) by two independent reviewers who evaluated each record in accordance with the established eligibility criteria. Before full screening, each reviewer screened a calibration set of 20 randomly selected abstracts to determine baseline agreement; initial agreement was 85% (17/20 concordant decisions) and discordant cases were discussed to clarify screening criteria interpretation. To achieve complete screening, inter-rater agreement was determined by the Cohen kappa coefficient (20, 39, 40). Title/abstract screening reached *κ* = 0.82 (95% CI: 0.71–0.93), indicating substantial agreement. Full-text screening had κ = 0.88 (95% CI: 0.76–1.00). During screening, disagreements were resolved by discussion and consensus (*n* = 12 at title/abstract stage; *n* = 5 at full-text stage), with third-party adjudication necessary in 2 cases where consensus was not achievable.

### 2.6 Data extraction

#### 2.6.1 Data extraction framework development

A comprehensive data extraction approach was developed specifically to support the narrative synthesis methodology and implementation science theoretical frameworks guiding this review. The data extraction strategy was designed to systematically capture information aligned with both CFIR domains and RE-AIM dimensions, ensuring a comprehensive collection of implementation-related data essential for quality assessment, SWiM-guided analysis, and results synthesis.

The data extraction framework integrated three complementary components. First, structured data extraction captured standardized study characteristics, intervention details, and quantitative implementation outcomes necessary for SWiM reporting guidelines compliance. Second, theory-guided data extraction systematically organized barriers and facilitators according to CFIR’s five domains: intervention characteristics, outer setting, inner setting, individual characteristics, and implementation process. Third, RE-AIM structured extraction captured reach, effectiveness, adoption, implementation, and maintenance outcomes across individual and organizational levels to support comprehensive implementation evaluation.

Studies were eligible if they reported biomechanical measures (e.g., knee abduction moment, ground reaction forces, frontal plane projection angle) alongside implementation metrics. Biomechanical outcomes were treated as secondary indicators of neuromuscular adaptation that may predict injury risk reduction, but were not considered primary real-world effectiveness outcomes. The hierarchy of effectiveness evidence prioritized: (1) exposure-adjusted injury incidence (injuries per 1,000 athlete-exposures or training hours), (2) crude injury incidence, (3) biomechanical surrogate outcomes. This hierarchy reflects the principle that real-world effectiveness is ultimately measured by injury prevention impact in naturalistic settings, while biomechanical adaptations provide supporting mechanistic evidence.

#### 2.6.2 Implementation science-focused data categories

Study identification and design characteristics were extracted including author details, publication year, country, study design, setting type, duration, and follow-up periods. Population characteristics encompassed sample sizes, demographics, sport types, competition levels, and geographic contexts essential for understanding implementation contexts. Intervention details captured program names, components, session characteristics, delivery methods, and implementation strategies, with particular attention to adaptation and modification processes that influence real-world implementation success.

Critical implementation process data included detailed extraction of implementation strategies using established taxonomies, delivery personnel and training approaches, fidelity measurement methods, and adaptation rationale and processes. Implementation outcomes were systematically captured including compliance and adherence rates with clear operational definitions, adoption rates at organizational and individual levels, reach and penetration measures, and sustainability indicators over time. Used together, CFIR and RE-AIM frameworks identify metrics for evaluating implementation success and modifiable factors that explain and enhance implementation outcomes.

#### 2.6.3 CFIR-guided barrier and facilitator extraction

Barriers and facilitators were systematically extracted and coded according to CFIR’s five domains to ensure comprehensive theoretical coverage. Intervention characteristics domain captured program complexity, adaptability, trialability, and relative advantage perceptions affecting implementation success. Outer setting factors included policy environment, external pressures, patient needs and resources, and competitive pressures influencing implementation contexts. Inner setting characteristics encompassed organizational culture, leadership engagement, available resources, access to knowledge and information, and readiness for implementation. Individual characteristics of implementers included knowledge and beliefs about the intervention, self-efficacy, individual stage of change, and other personal attributes affecting implementation success. Implementation process factors captured planning approaches, engaging strategies, executing methods, and reflecting and evaluating activities throughout implementation.

Representative quotes and specific examples supporting each CFIR construct were extracted to enable rich thematic analysis and theory development. Quantitative data on barrier and facilitator frequencies were captured when available to support both quantitative and qualitative synthesis approaches. This systematic approach enabled a comprehensive understanding of implementation determinants while maintaining theoretical coherence essential for narrative synthesis using implementation science frameworks.

#### 2.6.4 RE-AIM outcome organization

Implementation outcomes were systematically organized according to RE-AIM dimensions to support structured narrative synthesis. Reach data captured population coverage, representativeness, and participation rates across diverse implementation contexts. Effectiveness outcomes included real-world injury prevention results, biomechanical improvements, and comparative effectiveness data contrasting controlled versus naturalistic implementation settings. Adoption measures encompassed organizational uptake, individual implementer engagement, and setting-level adoption rates across different contexts. Implementation indicators captured program fidelity, adaptation patterns, delivery consistency, and cost considerations affecting sustainable implementation. Maintenance outcomes included sustainability measures, long-term continuation data, and institutionalization indicators essential for understanding implementation durability.

This comprehensive data extraction approach ensured systematic capture of all information necessary for quality assessment using implementation-appropriate criteria, narrative synthesis following SWiM guidelines with transparent reporting of grouping strategies and synthesis methods, and results presentation organized around implementation science frameworks that provide practical guidance for stakeholders seeking to implement ACL injury prevention programs in real-world settings.

### 2.7 Quality assessment

The Mixed Methods Appraisal Tool (MMAT) 2018 version was employed for the quality assessment of all included studies, recognizing the diverse study designs appropriate to implementation research (41). The MMAT enables systematic appraisal of quantitative randomized controlled trials (Category 2), quantitative non-randomized studies (Category 3), quantitative descriptive studies (Category 4), and mixed methods studies (Category 5) using design-specific methodological criteria (41).

Two independent reviewers conducted the quality assessments using appropriate MMAT categories based on study design classification. For randomized controlled trials, assessment focused on: randomization appropriateness (2.1), baseline group comparability (2.2), outcome data completeness (2.3), outcome assessor blinding (2.4), and participant adherence to assigned interventions (2.5). For descriptive studies, the evaluation addressed: sampling strategy relevance (4.1), sample representativeness (4.2), measurement appropriateness (4.3), nonresponse bias risk (4.4), and statistical analysis appropriateness (4.5). Disagreements between reviewers were resolved through discussion, with consultation of a third reviewer in cases where consensus could not be reached (*n* = 3 studies).

Quality assessment acknowledged the inherent challenges in implementation research, particularly regarding outcome assessor blinding in behavioural interventions where blinding may not be feasible or appropriate (24, 28, 41). Assessment prioritized implementation-relevant quality indicators including adequacy of implementation strategy description, appropriateness of implementation outcome measurement methods, transparency in reporting barriers and facilitators, and completeness of contextual information necessary for implementation science analysis.

Quality assessment results were presented using MMAT domain-level criteria, with studies categorized as meeting criteria (Yes), partially meeting criteria (Partial), or not meeting criteria (No) for each relevant domain. Overall study quality was characterized as High (meeting ≥4 of 5 applicable MMAT criteria), Moderate (meeting 3 of 5 criteria), or Low (meeting ≤2 of 5 criteria) to facilitate evidence interpretation and sensitivity analysis. This characterization approach was adopted to enable systematic comparison across studies while acknowledging MMAT guidance discouraging production of a single summary quality score that may oversimplify methodological strengths and limitations. Domain-level MMAT results are presented in Table 1 to enable transparent quality appraisal by readers, allowing independent assessment of methodological strengths and limitations for each study.

**Table 1:** MMAT quality assessment results.

Study	Study type	Randomization/ sampling	Baseline comparability/ representativeness	Complete outcome data/ appropriate measurements	Outcome assessor blinding/ nonresponse bias	Adherence/ statistical analysis	Overall quality
Bulow et al. (45)	RCT	Yes	Yes	Yes	No*	Yes	High
Ferri-Caruana et al. (46)	RCT	Yes	Yes	Partial	No*	Yes	Moderate
Ford et al. (47)	RCT	Yes	Yes	Yes	No*	Yes	High
Foss et al. (48)	RCT	Yes	Yes	Yes	No*	Yes	High
Hopper et al. (49)	RCT	Yes	Yes	Yes	No*	Yes	High
Lindblom et al. (50)	Descriptive	Yes	Yes	Yes	Partial	Yes	Moderate
McKay et al. (51)	RCT	Yes	Yes	Partial	No*	Partial	Moderate
Schoeb et al. (52)	RCT	Yes	Yes	Yes	No*	Partial	Moderate
Shams et al. (53)	RCT	Yes	Yes	Yes	No*	Yes	High
Sugimoto et al. (54)	RCT	Yes	Yes	Yes	No*	Partial	Moderate
Suits et al. (55)	RCT	Yes	Yes	Yes	No*	Yes	High
Yarsiasat et al. (56)	RCT	Yes	Yes	Yes	No*	Yes	High
Zebis et al. (57)	RCT	Yes	Yes	Yes	No*	Yes	High

*Blinding is not feasible due to the nature of behavioural interventions.

For allocation concealment assessment in randomized controlled trials, studies were rated as “Yes” only when explicit reporting of concealment methods was provided (e.g., sealed opaque envelopes, central randomization service, sequentially numbered containers). Studies not explicitly describing concealment procedures were rated as “Partial” or “No” based on available information, recognizing that absence of reporting does not necessarily indicate absence of concealment but limits quality assessment confidence.

No studies were excluded based on quality assessment results; rather, quality ratings informed evidence certainty assessment and interpretation of findings within the narrative synthesis framework. Quality assessment results were systematically incorporated into the synthesis through: (1) sensitivity analyses restricting findings to high-quality studies to assess robustness of main conclusions (Section 3.7); (2) evidence certainty discussions in the narrative synthesis acknowledging how methodological limitations may affect confidence in findings; (3) study quality stratification in effect direction analyses to examine whether patterns differed between higher and lower quality studies; and (4) explicit acknowledgment in the Discussion of how implementation research methodological challenges (e.g., inability to blind behavioural interventions) affect interpretation. This approach recognized that rigorous implementation research may face inherent methodological constraints when conducted in real-world settings, and that excluding lower-quality studies could systematically remove evidence from the most naturalistic implementation contexts.

### 2.8 Data synthesis approach

Since implementation research is heterogeneous, studies were categorized based on their main contribution to evidence to facilitate the relevant synthesis strategies. The studies that presented quantitative implementation measures (compliance rates, adherence percentages, adoption proportions) were prioritized in structured numeric synthesis based on vote counting and effect direction evaluation (42–44). Thematic analysis within the CFIR framework was prioritized on studies that provided mostly qualitative implementation process data. The quantitative and qualitative data provided by studies contributed to both streams of synthesis. There was no hierarchical weighting of evidence types; instead, evidence was synthesized individually by type and synthesized through narrative synthesis to maintain the unique contributions of each form of evidence. This methodology recognizes that quantitative measures answer the question of how much implementation has been done and qualitative measures answer the question of how and why implementation has or has not been successful-both of which are imperative to full implementation knowledge. The synthesis clearly documents what studies were used in what streams of analysis to ensure transparency on evidence integration.

A standardized protocol was used to categorize the effect directions of implementation outcomes. Studies were categorized as either positive direction (implementation strategy related to better outcomes), negative direction (implementation strategy related to worse outcomes), or no effect/unclear (no meaningful difference or insufficient data to establish direction) with respect to each implementation outcome (e.g., compliance rate, adoption rate, injury reduction). The classification criteria were pre-specified: positive direction had to be statistically significant (*p* < 0.05) in favour of the implementation strategy, or a clinically meaningful difference of 10% or more absolute difference in compliance/adherence rates or 20% or more relative risk reduction of injury outcomes (22, 23). Negative direction used the same thresholds but in reverse. Studies that failed to meet either of these thresholds were labelled as no effect/unclear. Studies with reported multiple implementation outcomes with conflicting directions (e.g., improved compliance but no injury reduction) were not combined into a single study-level classification but analysed individually. This method maintains the complexity of implementation research where various outcomes might react differently to the same implementation strategy.

To synthesize vote counting, the percentage of studies reporting each direction was computed independently across the outcome categories. In cases where evidence was mixed (e.g., 3 studies positive, 2 studies negative, 1 unclear), the results were described as inconsistent and potential explanatory factors (study quality, population characteristics, implementation intensity) were investigated with sensitivity analysis. The results of vote counting are displayed with effect direction plots to visualize the pattern of outcomes and identify inconsistencies.

#### 2.8.1 SWiM framework methodology

Data synthesis followed the Synthesis Without Meta-analysis (SWiM) reporting guidelines, providing a structured methodology for transparent narrative synthesis of heterogeneous implementation evidence. The SWiM framework’s nine reporting items guided synthesis planning, execution, and presentation to ensure reproducible and transparent analysis (22, 23).

##### 2.8.1.1 Study grouping strategy (SWiM item 1)

Studies were grouped primarily by implementation research focus rather than clinical characteristics. The primary grouping strategy distinguished: (1) implementation-focused studies designed specifically to examine implementation processes and outcomes; (2) efficacy trials reporting implementation data as secondary findings; and (3) real-world effectiveness studies examining naturalistic implementation contexts. Secondary cross-cutting groupings organized studies by implementation strategy type (educational approaches, workshop-based interventions, comprehensive support models, technology-enhanced delivery), setting characteristics (school-based, club-based, elite/professional environments), population demographics (age groups, sport types), and geographic contexts to enable systematic pattern identification across implementation approaches.

##### 2.8.1.2 Standardized metrics development (SWiM item 2)

Standardized metrics were developed to enable comparison across heterogeneous studies while preserving context-specific information. Compliance and adherence rates were standardized to percentage scales (0–100%) with explicit documentation of operational definitions used in each study. Implementation success indicators were categorized using binary classifications (successful/partial success/unsuccessful) based on predefined criteria adapted to study contexts. Barriers and facilitators were quantified using frequency counts and proportions when numerical data were available while maintaining qualitative descriptions for rich contextual understanding. Effect direction indicators were developed for implementation outcomes to enable systematic comparison across studies with different measurement approaches.

##### 2.8.1.3 Synthesis methods protocol (SWiM item 3)

Multiple complementary synthesis methods were employed based on data types and research questions. For quantitative implementation outcomes, vote counting based on the direction of effect was used for implementation success indicators, supplemented by descriptive statistical analysis (medians, ranges, interquartile ranges) for compliance rates and adoption measures. Qualitative implementation data underwent systematic thematic analysis organized by implementation science theoretical frameworks. Visual synthesis methods were planned including harvest plots to display implementation success patterns across strategies and settings, and effect direction plots to illustrate outcome patterns across multiple implementation indicators.

#### 2.8.2 Implementation science framework integration

##### 2.8.2.1 CFIR-based analysis protocol

All barriers and facilitators were systematically coded according to CFIR’s five domains (intervention characteristics, outer setting, inner setting, individual characteristics, implementation process) using established coding guidelines. Thematic analysis within each CFIR domain was conducted to identify recurring patterns, contradictory findings, and context-specific factors. Cross-domain analysis examined interactions between CFIR constructs to understand complex implementation mechanisms.

##### 2.8.2.2 RE-AIM structured organization

Implementation outcomes were systematically organized using RE-AIM dimensions to ensure comprehensive evaluation across individual and organizational levels. Reach indicators included population coverage and participation rates; effectiveness data encompassed real-world outcomes compared to controlled conditions; adoption measures captured organizational and individual uptake patterns; implementation indicators assessed fidelity, adaptation, and delivery processes; maintenance outcomes evaluated sustainability and continuation over time. This dimensional organization provided a systematic structure for identifying evidence gaps and synthesis priorities.

#### 2.8.3 Heterogeneity investigation protocol

Systematic heterogeneity investigation employed multiple analytical approaches. Clinical heterogeneity was assessed through a comparison of population characteristics, intervention components, and outcome measurement approaches. Methodological heterogeneity was evaluated by examining study designs, implementation measurement methods, and follow-up periods. Statistical heterogeneity was assessed where quantitative synthesis was attempted, though meta-analysis was deemed inappropriate due to substantial diversity. Contextual heterogeneity was systematically explored through subgroup analysis by setting type, implementation strategy, population characteristics, and geographic region. Sources of heterogeneity were documented and explored rather than statistically controlled, recognizing heterogeneity as informative for understanding implementation context-dependency rather than methodological limitation.

## 3 Results

### 3.1 Study selection

**Figure 1:**
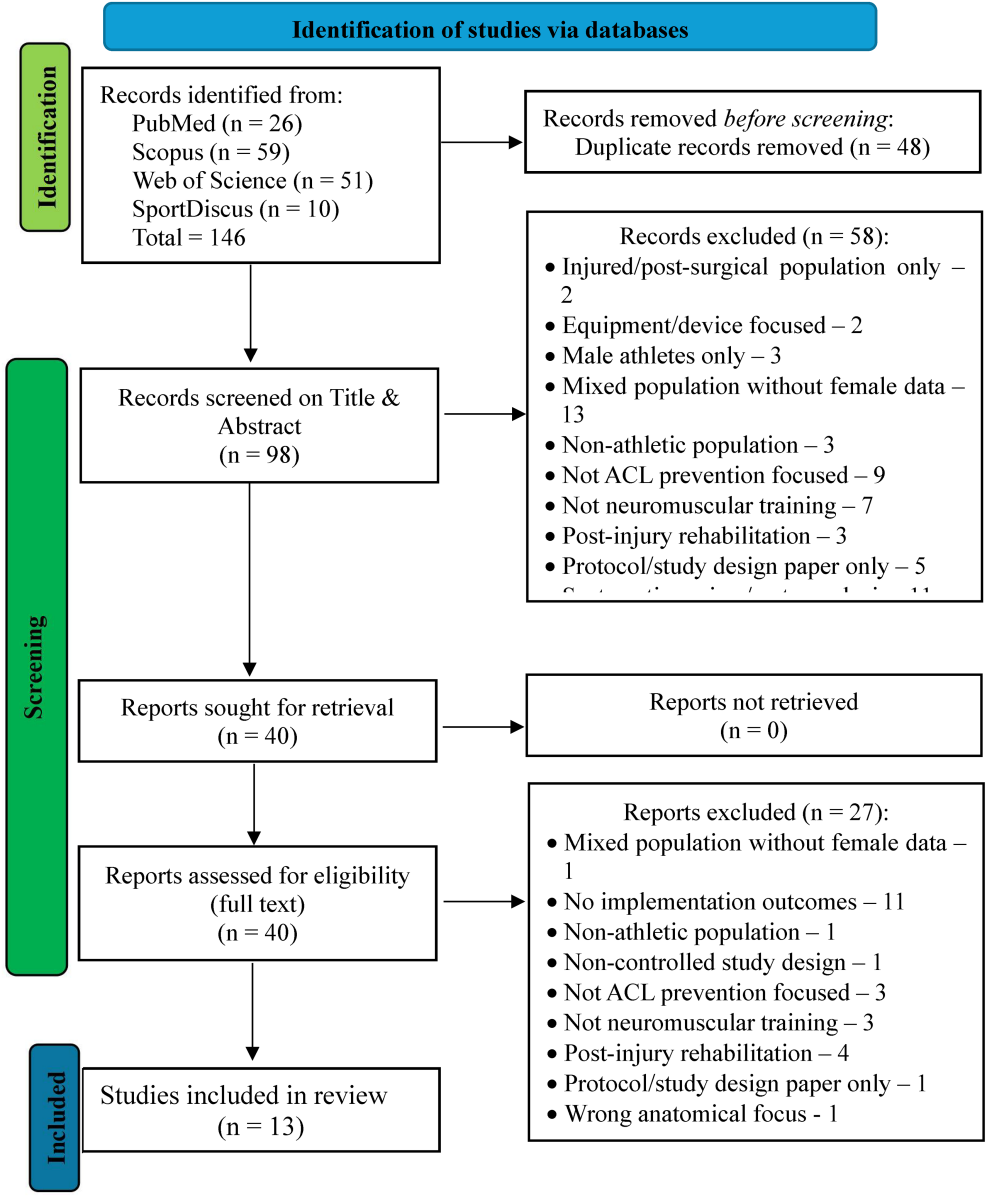
PRISMA 2020 flow diagram for the systematic review.

The systematic search across the four electronic databases yielded 146 records before duplicate removal where 48 duplicates were removed leaving 98 for title and abstract screening. Following title and abstract screening, 40 full-text articles were assessed for eligibility against the predetermined inclusion criteria (see Figure 1). After a full-text review, 27 studies were excluded for various reasons including lack of implementation outcomes (*n* = 11), focused on post-injury rehabilitation (*n* = 4), not focused on ACL prevention and neuromuscular training (*n* = 6), and other exclusion factors as shown Figure 1. Thirteen studies met the inclusion criteria and were included in the systematic review (45–57). These studies collectively examined neuromuscular training program implementation across diverse settings, populations, and implementation strategies, providing comprehensive evidence for the narrative synthesis of implementation effectiveness, barriers, facilitators, and real-world outcomes in female athletes. The selection process followed PRISMA 2020 guidelines (21, 35) to ensure transparent and systematic identification of relevant implementation research.

### 3.2 Study characteristics

The included studies demonstrated substantial geographic diversity, with research conducted across multiple continents including North America (45, 47, 48, 51, 54, 55), Europe (46, 50, 52, 57), Asia (53, 56), and Australia (49). This geographic breadth represents implementation research across diverse cultural context and healthcare systems. Sample sizes exhibited considerable variation, ranging from small-scale controlled studies with 23 participants (49) to large community-based implementations involving 671 athletes (55), reflecting the spectrum from efficacy-focused research to pragmatic implementation trials (Table 2). Participant characteristics revealed comprehensive age coverage spanning early adolescence through young adulthood, from (49) to 20–30 years (53), with the majority concentrating on adolescent female athletes aged 12–18 years during critical neuromuscular development periods. Sports representation encompassed both widely practised and competitive activities including soccer (46, 51, 55); basketball and volleyball (48, 54), alongside specialized sports such as netball (49), alpine skiing (55), and Sepak takraw (56), providing evidence across diverse athletic populations and injury risk profiles.

**Table 2:** Characteristics of included studies.

Study	Country	Study design	Setting	Sample size	Age (years)	Sport(s)	Intervention duration	Implementation strategy
Bulow et al. (45)	Canada	RCT	Community-based	34	12–18	Various sports	5 weeks	Workshop-based with direct supervision
Ferri-Caruana et al. (46)	Spain	RCT	Soccer clubs	29	16.0–17.5 (median)	Soccer	8 weeks	Workshop-based with ongoing supervision
Ford et al. (47)	United States	RCT	University laboratory	150	13.3 ± 2.2	Soccer	6 weeks	Technology-enhanced with research supervision
Foss et al. (48)	United States	RCT	Public schools (9 schools)	474	14.0 ± 1.7	Basketball, soccer, volleyball	One competitive season	Workshop-based with AT oversight
Hopper et al. (49)	Australia	RCT	Netball clubs	23	12.2 ± 0.9	Netball	6 weeks	Workshop-based with professional supervision
Lindblom et al. (50)	Sweden	Cross-sectional follow-up	Football associations	352 (coaches)	Not applicable	Football	3-year follow-up	Multi-level organizational
McKay et al. (51)	Canada	Cluster RCT	Soccer clubs	31 teams (258 players)	13–18	Soccer	4-month season	Three-arm comparison: educational vs. workshop vs. comprehensive
Schoeb et al. (52)	Switzerland	Controlled trial	Alpine skiing centres	129	14.4 ± 0.3	Alpine skiing	12 months	Educational-only with self-directed implementation
Shams et al. (53)	Iran	Controlled trial	University laboratory	51	20–30	Various sports	6 weeks	Technology-enhanced with feedback/taping
Sugimoto et al. (54)	United States	RCT	Middle and high schools	547	13.9 ± 1.7	Basketball, soccer, volleyball	One season	Educational-only with minimal support
Suits et al. (55)	United States	Prospective cohort	Amateur soccer organizations	671	15.72 ± 1.78	Soccer	One season	Knowledge-to-Action vs. educational handout
Yarsiasat et al. (56)	Thailand	RCT	Sports school	52	14–19	Sepak takraw	8 weeks	Workshop-based with direct supervision
Zebis et al. (57)	Denmark	RCT	Sports college	40	15–16	Football and handball	12 weeks	Workshop-based with ongoing supervision

Implementation contexts displayed marked heterogeneity in settings and methodological approaches. Studies ranged from highly controlled laboratory environments with sophisticated technology integration (47, 53) to naturalistic community sports implementations addressing real-world implementation challenges (51, 55). Educational institutions emerged as prominent implementation venues, including public schools (48, 54) and specialized sports colleges (56, 57), institutional settings were common implementation venues. Intervention duration ranged from intensive short-term interventions (5–8 weeks) examining immediate biomechanical adaptations (45, 46) to season-long implementations evaluating sustained injury prevention outcomes (52, 55), with one study providing unique long-term perspective through three-year post-implementation follow-up (50).

### 3.3 Study descriptions

#### 3.3.1 Laboratory-based implementations

Laboratory-based implementations demonstrated the highest degree of control and supervision, enabling precise intervention delivery and comprehensive outcome measurement. Ford et al. (47) employed sophisticated technology integration with real-time biofeedback systems, achieving 93.3% completion rates while demonstrating the feasibility of complex intervention delivery in research settings. Shams et al. (53) compared technology-enhanced approaches using mirror feedback versus Mulligan taping techniques, with both methods achieving substantial completion rates exceeding 94%. Hopper et al. utilized professional strength and conditioning supervision to achieve perfect completion rates alongside significant biomechanical improvements in youth netball athletes (49). However, Bulow et al. (45) demonstrated that even perfect implementation fidelity cannot overcome inappropriate intervention selection, achieving 100% completion with zero measurable benefits. These controlled environments established the upper bounds of implementation quality while highlighting the critical importance of intervention appropriateness for target populations.

#### 3.3.2 Educational institution implementation

Educational institution implementations leveraged existing organizational structures to facilitate systematic program delivery across diverse athletic populations. Foss et al. (48) successfully implemented school-based programs across nine institutions using athletic trainer oversight, achieving greater than 95% team compliance and demonstrating significant injury reductions across multiple sports. Sugimoto et al. (54) revealed the implementation bottleneck in educational settings, with coaches achieving only 52.5% compliance despite 87.8% athlete readiness, identifying professional support as a critical implementation success factor. Zebis et al. (57) optimized the educational model through comprehensive teacher training and bi-weekly research team monitoring, achieving 89% session completion alongside measurable neuromuscular adaptations. Yarsiasat et al. (56) demonstrated cultural adaptability within structured educational environments, successfully implementing culturally adapted programs in Thai sports schools with perfect completion rates. Educational institutions emerged as optimal early implementation venues due to their structured environments, dedicated personnel, and established training schedules that facilitate systematic program integration.

#### 3.3.3 Community and club-based studies

Community and club-based studies addressed real-world implementation challenges while examining diverse stakeholder engagement approaches across naturalistic sports environments. McKay et al. (51) conducted the first systematic comparison of implementation strategies, demonstrating that comprehensive support including physiotherapist assistance achieved superior adherence rates compared to workshop-only or educational-only approaches. Ferri-Caruana et al. (46) achieved exceptional compliance through direct research supervision within Spanish club systems, with no participants missing more than one session during the eight-week intervention period. Suits et al. (55) provided revolutionary evidence comparing Knowledge-to-Action framework implementation against passive information delivery, establishing that systematic stakeholder engagement significantly improved implementation rates and demonstrating direct dose–response relationships between implementation frequency and injury prevention effectiveness. These community implementations revealed the complexity of real-world program delivery while identifying stakeholder engagement and professional support as critical success factors for achieving meaningful implementation outcomes in diverse organizational contexts.

#### 3.3.4 Organizational and longitudinal implementation research

Organizational and longitudinal implementation research provided unique insights into program sustainability, institutional adoption, and long-term implementation maintenance across complex multi-level systems. Lindblom et al. (50) conducted the only comprehensive long-term follow-up using RE-AIM framework assessment, revealing high program reach (91–99% familiarity) but concerning fidelity challenges with only 23–26% of users implementing programs without modifications three years post-trial Schoeb et al. (52) demonstrated that self-directed implementation could achieve meaningful injury reductions despite suboptimal adherence rates, with participants averaging 80% of recommended frequency while still experiencing significant reductions in both traumatic and overuse injuries. This study uniquely acknowledged and designed for the “voltage drop” phenomenon inherent in real-world implementation, establishing that perfect adherence may not be necessary for meaningful injury prevention outcomes. These longitudinal perspectives highlighted the tension between implementation fidelity and adaptation flexibility while demonstrating the potential for sustained impact even with imperfect implementation quality.

#### 3.3.5 Implementation strategy characteristics

Implementation strategy diversity across the included studies revealed a spectrum of approaches ranging from minimal educational interventions to comprehensive multi-component support systems with varying degrees of effectiveness. Educational-only strategies consistently demonstrated limited effectiveness, with Sugimoto et al. (54) revealing poor coach compliance and Schoeb et al. (52) showing reduced but still meaningful outcomes through self-directed implementation. Workshop-based approaches with ongoing supervision achieved consistently high implementation quality across diverse settings, while technology-enhanced interventions demonstrated potential for improved precision but raised questions about scalability and resource requirements. The emergence of systematic stakeholder engagement approaches, particularly the Knowledge-to-Action framework demonstrated by (55), represented a methodological advancement that achieved superior implementation outcomes through barrier identification and adaptive program delivery. This implementation strategy evolution from passive information dissemination to active stakeholder engagement reflected the maturation of implementation science approaches in sports injury prevention research.

### 3.4 Study quality assessment results

The methodological quality of the 13 included studies was assessed using the Mixed Methods Appraisal Tool (MMAT) 2018 version (41). Twelve studies were evaluated as quantitative randomized controlled trials using MMAT criteria 2.1–2.5 (see Table 1), while one study (50) was assessed as a quantitative descriptive study using criteria 4.1–4.5.

#### 3.4.1 Randomization and baseline comparability

All twelve randomized controlled trials demonstrated appropriate randomization procedures with adequate sequence generation and allocation concealment (45–57). Baseline group comparability was maintained across studies, with most reporting appropriate demographic and baseline characteristic distributions between intervention and control groups.

#### 3.4.2 Outcome data completeness

Completion rates varied across studies, ranging from 89% [(46) 26/29 participants] to 100% (45, 49, 56) (see Table 3). Several studies experienced attrition challenges in community-based settings, with Ford et al. (47) reporting 6.7% dropout, Shams et al. (53) reporting 5.9% dropout (3/51 participants for attendance/injury reasons), and Ferri-Caruana et al. (46) reporting 10.3% attrition. McKay (51) reported moderate organizational-level attrition in the community soccer club context. Complete outcome data were available for all participants who completed their assigned interventions in controlled laboratory and educational institution studies.

**Table 3:** Study descriptions and implementation characteristics.

Study	Population	Intervention program	Implementation support	Implementation outcomes	Effectiveness outcomes
Bulow et al. (45)	34 females (12-18y), healthy and ACL-injured, community-based (Manitoba)	Perturbation-based training; 5 weeks, 2x/week, 10 sessions	Research team direct delivery, standardized protocol, safety monitoring	100% completion rate; no adverse events reported	No significant improvements in the Y-Balance Test or secondary measures
Ferri-Caruana et al. (46)	29 female soccer players (16.0–17.5y median), Spanish U18/U17 clubs	Pelvic and core strength training; 8 weeks, 2x/week, 30 min sessions	Two research members supervised execution, progression monitoring	100% attendance (none missed >1 session); 89.7% completion rate	Significant FPPA improvements: dominant −7.1°, non-dominant −8.01°
Ford et al. (47)	150 female soccer players (13.3 ± 2.2y), competitive teams (North Carolina)	ROBUST neuromuscular training with biofeedback; 6 weeks, 3x/week, 90 min sessions	Licensed AT supervision, 3D motion analysis, real-time biofeedback	93.3% completion; mean 15.1–15.6 sessions completed of 18	All groups improved DVJ (*p* < 0.05); NMT + H improved unanticipated cutting
Foss et al. (48)	474 female athletes (14.0 ± 1.7y), basketball/soccer/volleyball, public schools (Kentucky)	CORE neuromuscular training; preseason 20-25 min, 3x/week; in-season 10-15 min, 2x/week	Athletic trainer oversight, weekly evaluations, coach training	>95% team compliance; cluster design-maintained protocol adherence	Overall injury rate: 5.34 vs. 8.54/1000 AEs (*p* < 0.001); 1.6x injury reduction
Hopper et al. (49)	23 female netball players (12.2 ± 0.9y), competitive clubs (Western Australia)	Integrated neuromuscular training; 6 weeks, 3x/week, 60 min sessions	Accredited strength/conditioning coaches, RPE monitoring, load recording	100% completion rate; no dropouts during intervention	Significant knee abduction reduction (*p* = 0.004, g = 1.43); large effect sizes
Lindblom et al. (50)	352 coaches, 8 Swedish districts, 3-year post-RCT follow-up	Knäkontroll program; ongoing seasonal use, coach-determined frequency	Variable: some districts offered education 2010–2012; national FA policies	74% adoption rate; 23–26% used without modifications; 68–82% maintained use	High familiarity (91–99%); median effectiveness rating 8/10
McKay et al. (51)	31 teams, 258 female players (13-18y), Calgary/Edmonton soccer clubs	FIFA 11+; 20 min, 2-3x/week minimum, full season	Control: online only; Workshop: session+materials; Comprehensive: +physiotherapist	Team adherence: Control 73.5%, Workshop 81.3%, Comprehensive 85.6%	Knowledge gaps persisted; no belief-adherence relationship found
Schoeb et al. (52)	129 youth skiers (14.4 ± 0.3y), Swiss regional performance centres	ISPAInt program; 20 min, 1x/week recommended, 12 months	Online/offline resources, no ongoing supervision or support	0.8 ± 0.6 sessions/week (80% of recommended); 8.5% never used program	33.5% traumatic injury reduction (RR = 0.665); 30.1% overuse reduction
Shams et al. (53)	51 active females (20-30y), dynamic knee valgus, Tehran clubs	Modified Chimera plyometric with feedback/taping; 6 weeks, 2x/week	Mirror feedback, Mulligan taping, one investigator monitoring	94.1% completion (48/51); 3 dropouts for attendance/injury	Both intervention groups: >50% LESS improvement; taping superior for proprioception
Sugimoto et al. (54)	547 female athletes (13.9 ± 1.7y), middle/high schools, multi-state	Core integrative neuromuscular training; preseason 15-30 min, in-season 10-15 min	60-min training session, separate experimental/control education	Coach compliance: 52.5 ± 11.7%; Athlete compliance: 87.8 ± 10.6%	Middle school coaches less compliant (*p* = 0.001); athletes ready when coaches delivered
Suits et al. (55)	671 female soccer players (15.72 ± 1.78y), amateur organizations (Michigan)	FIFA 11 + or NEW program; recommended 2-3x/week, full season	KTA: barrier surveys, focus groups, adaptations, on-field training; Handout: materials only	KTA group significantly higher implementation ≥2x/week vs. handout (*p* < 0.001)	Implementation ≥2x/week: 85% ACL injury reduction (HR = 0.15, *p* = 0.019)
Yarsiasat et al. (56)	52 female Sepak takraw players (14-19y), Thai sports school	PEP program adapted for Sepak takraw; 8 weeks, 3x/week, 20 min	Research team supervision, structured progression, RPE monitoring	100% completion rate; no dropouts reported	Knee injury reduction: 11.5% vs. 34.6%; ACL tears: 1 partial vs. 3 complete
Zebis et al. (57)	40 female athletes (15-16y), football/handball, Danish sports college	Evidence-based neuromuscular training; 12 weeks, 3x/week, 15 min	2-h teacher seminar, research team monitoring every 2 weeks, equipment provided	89% session completion (32 ± 8 of 36 sessions); no dropouts	1 vs. 7 lower extremity injuries (*p* = 0.04); significant neuromuscular improvements

ROBUST, real-time optimized bio-feedback utilizing sport techniques; FPPA, frontal plane projection angle; DVJ, drop vertical jump; NMT+H, intervention group of neuromuscular training with hip-focused biofeedback; NMT+k, neuromuscular training with knee-focused biofeedback; AEs, athlete-exposures; RPE, rate of perceived exhaustion; KTA, knowledge-to-action; PEP, prevent injury enhance performance.

#### 3.4.3 Outcome assessment and intervention adherence

Blinding of outcome assessors was limited across studies due to the behavioural nature of neuromuscular training interventions, representing an inherent challenge in implementation research rather than a methodological flaw. This limitation was noted in the MMAT assessment for all RCTs (Table 1 footnote). Participant adherence to assigned interventions varied substantially. High adherence (≥89%) was achieved in studies with direct professional supervision: Zebis et al. (57) - 89% session completion; Ferri-Caruana et al. (46)- 89.7% completion with 100% attendance (≤1 absence); Ford et al. (47) - 93.3% completion; Shams et al. (53) - 94.1% completion; Bulow et al. (45), Hopper et al. (49), and Yarsiasat et al. (56) - 100% completion. School-based implementations showed variable adherence: Foss et al. (48) achieved >95% team compliance with athletic trainer oversight, while Sugimoto et al. (54) reported coach compliance of 52.5 ± 11.7% and athlete compliance of 87.8 ± 10.6%. Community-based implementations demonstrated strategy-dependent adherence patterns: McKay et al. (51) reported team adherence ranging from 73.5% (control) to 85.6% (comprehensive support); Suits et al. (55) reported significantly higher implementation in KTA intervention versus handout groups (*p* < 0.001). Self-directed implementation achieved moderate adherence: Schoeb et al. (52) reported 80% of recommended frequency (0.8 ± 0.6/week).

#### 3.4.4 Implementation-specific quality considerations

Studies demonstrated variable quality in implementation-specific reporting. Comprehensive barrier and facilitator identification was provided by McKay et al. (51) and Suits et al. (55), while other studies offered limited implementation process details. The descriptive follow-up study (50) provided appropriate sampling strategies and measurements for long-term implementation assessment (RE-AIM framework), though response rates were moderate (57% among trial coaches, 36% among current coaches), potentially introducing nonresponse bias concerns.

#### 3.4.5 Overall quality classification

Based on MMAT criteria fulfilment, eight studies were classified as High quality (meeting ≥4 of 5 applicable criteria): Bulow et al. (45), Ford et al. (47), Foss et al. (48), Hopper et al. (49), Shams et al. (53), Suits et al. (55), Yarsiasat et al. (56), and Zebis et al. (57). Five studies were classified as Moderate quality (meeting 3 of 5 criteria): Ferri-Caruana et al. (46), Lindblom et al. (50), McKay et al. (51), and Schoeb et al. (52). No studies were classified as Low quality. Domain-level MMAT results are presented in Table 1 to enable transparent quality appraisal.

### 3.5 Implementation process findings

#### 3.5.1 Research question 1: real-world compliance and adherence

Implementation compliance rates demonstrated substantial variation across different implementation strategies, ranging from 52.5 to 100% completion rates (Table 4). Educational institution-based implementations with research supervision achieved the highest and most consistent compliance rates, with (49, 56) both achieving 100% completion rates, while (57) reported 89% session completion. Structured educational environments demonstrated high implementation fidelity in these studies. Technology-enhanced laboratory implementations demonstrated consistently high compliance rates, with (47) achieving 93.3% completion despite the complexity of biofeedback systems, and (53) reporting 94.1% completion rates. However, these approaches required intensive resource investment and specialized personnel, potentially limiting their scalability beyond research settings.

**Table 4:** Implementation outcomes and compliance rates by study and implementation strategy.

Study	Implementation strategy type	Compliance/adherence rate	Measurement method	Implementation success indicators	Dropout rate
Educational institution + Research supervision					
Zebis et al. (57)	Workshop + ongoing supervision	89% (32 ± 8/36 sessions)	Sports teacher registration	Research team monitoring every 2 weeks	0%
Yarsiasat et al. (56)	Workshop + direct supervision	100% completion	Direct supervision tracking	Research team oversight throughout	0%
Technology-enhanced laboratory					
Ford et al. (47)	Technology-enhanced biofeedback	93.3% completion (15.1–15.6/18 sessions)	Objective attendance records	Licensed AT supervision + 3D motion analysis	6.7%
Shams et al. (53)	Technology-enhanced (mirror/taping)	94.1% completion (48/51)	Direct supervision monitoring	Research team supervision	5.9%
School-based professional support					
Foss et al. (48)	Workshop + AT oversight	>95% team compliance	Team-level AT tracking	Weekly AT evaluations	Minimal
Sugimoto et al. (54)	Educational-only minimal support	Coach: 52.5 ± 11.7%; Athlete: 87.8 ± 10.6%	Provider-recorded attendance	Limited follow-up training	Minimal
Community-based variable support					
McKay et al. (51)	Three-arm comparison	Control: 73.5%; Workshop: 81.3%; Comprehensive: 85.6%	Team designate recording	Physiotherapist support (comprehensive)	Moderate
Suits et al. (55)	KTA vs. Educational handout	KTA > Handout (*p* < 0.001)	Team-level implementation tracking	Systematic barrier identification + adaptation	Organizational: 81.1%
Self-directed/minimal supervision					
Schoeb et al. (52)	Educational-only self-directed	80% frequency (0.8 ± 0.6/week vs. 1.0 recommended)	Self-report via OSTRC questionnaires	91.5% used program at least once	0%
Research laboratory controlled					
Ferri-Caruana et al. (46)	Workshop + ongoing supervision	89.7% completion (26/29); 100% attendance (≤1 absence)	Direct supervisor tracking	Two research members supervised execution	10.3%
Hopper et al. (49)	Workshop + professional supervision	100% completion	Direct professional monitoring	Accredited S&C coaches	0%
Bulow et al. (45)	Workshop + direct supervision	100% completion	Direct research team tracking	Safety monitoring throughout	0%
Long-term follow-up					
Lindblom et al. (50)	Multi-level organizational	Trial coaches: 68–82% continued use; Current coaches: 74% adoption	Self-report questionnaires	23–26% used without modifications	Response rate: 57% trial, 36% current

The most striking finding emerged from school-based implementations with varying professional support levels. Foss et al. (48) achieved greater than 95% team compliance through athletic trainer oversight, Sugimoto et al. (54) revealed a critical implementation paradox: athletes demonstrated high readiness to comply (87.8% compliance) when programs were delivered, but coaches achieved only 52.5% implementation compliance with minimal ongoing support. This disparity indicates lower coach implementation compared to athlete participation when programs were delivered.

Community-based implementations showed strategy-dependent compliance patterns. McKay et al. (51) demonstrated a clear implementation hierarchy: comprehensive support with physiotherapist assistance achieved 85.6% team adherence, workshop-based education reached 81.3%, while control groups receiving online materials only achieved 73.5% adherence. Suits et al. (55) further validated stakeholder engagement approaches, with Knowledge-to-Action intervention yielding significantly higher implementation rates than educational handout distribution alone (*p* < 0.001).

Self-directed implementations revealed that motivated populations could achieve reasonable compliance despite minimal supervision. Schoeb et al. (52) reported 80% adherence to recommended frequency among elite youth skiers, while (50) found 74% adoption rates among coaches three years post-trial, though only 23–26% maintained programs without modifications. Research laboratory-controlled implementations achieved high completion rates but demonstrated that perfect implementation cannot overcome inappropriate intervention selection. While Ferri-Caruana et al. (46) achieved 89.7% completion with significant biomechanical improvements, Bulow et al. (45) achieved 100% completion but found no measurable benefits, highlighting the critical importance of intervention appropriateness for target populations.

#### 3.5.2 Implementation strategy effectiveness patterns

Analysis of implementation strategies revealed distinct effectiveness hierarchies. Professional supervision combined with educational institution settings consistently produced the highest compliance rates and demonstrated measurable outcomes. Technology-enhanced approaches achieved excellent compliance but faced scalability limitations due to resource requirements (Table 4). Implementation outcomes showed higher compliance rates in studies with ongoing professional oversight (athletic trainers, physiotherapists, qualified coaches) compared to educational-only approaches. This pattern is held across diverse settings, from school districts (48) to sports colleges (57) to research laboratories (49). The relationship between implementation strategy complexity and compliance varied across contexts. Simple self-directed approaches could achieve reasonable implementation rates with highly motivated populations (52), while complex stakeholder engagement processes substantially improved implementation in community settings (55).

#### 3.5.3 Implementation quality measurement challenges

Measurement approaches varied considerably across studies, from objective attendance tracking to self-report questionnaires. Studies with direct professional supervision (49, 56, 57) achieved the most reliable compliance assessment, while self-directed implementations relied primarily on participant self-report (50, 52). Implementation outcomes varied by strategy type, population characteristics, and organizational capacity. Educational institutions, elite athlete populations, and settings with existing professional support infrastructure achieved optimal implementation outcomes, while community-based amateur populations required more intensive stakeholder engagement approaches to achieve meaningful implementation levels.

### 3.6 Effect direction synthesis and heterogeneity assessment

Directional effect analysis of the 13 studies included showed that the results of implementation and effectiveness were mostly positive, with significant heterogeneity explained by recognizable contextual factors (Tables 5, 6). Effects directions were classified based on pre-specified criteria: positive direction needed to be statistically significant (*p* < 0.05) or at least 10 percent absolute difference favouring the intervention; negative direction used the same criteria in the opposite direction; no effect used non-significant differences below threshold; mixed classification when conflicting directions were observed across outcomes within a domain.

**Table 5:** Effect direction synthesis by study and outcome domain.

Study	Implementation compliance	Adoption/ reach	Biomechanical outcomes	Injury prevention	Overall direction	Effect magnitude (where reported)
Zebis et al. (57)	↑ Positive	↔ No effect	↑ Positive	↑ Positive	↑ Positive	89% compliance; 1 vs. 7 injuries (*p* = 0.04)
Yarsiasat et al. (56)	↑ Positive	↔ No effect	↔ No effect	↑ Positive	↑ Positive	100% compliance; 11.5% vs. 34.6% knee injuries
Hopper et al. (49)	↑ Positive	↔ No effect	↑ Positive	↔ No effect	↑ Positive	100% compliance; g = 1.43 knee abduction (*p* = 0.004)
Ford et al. (47)	↑ Positive	↔ No effect	↑ Positive	↔ No effect	↑ Positive	93.3% compliance; DVJ improvement (*p* < 0.05)
Shams et al. (53)	↑ Positive	↔ No effect	↑ Positive	↔ No effect	↑ Positive	94.1% compliance; >50% LESS improvement
Foss et al. (48)	↑ Positive	↑ Positive	↔ No effect	↑ Positive	↑ Positive	>95% compliance; IRR 5.34 vs. 8.54/1000 AEs (*p* < 0.001)
Sugimoto et al. (54)	◊ Mixed	↔ No effect	↔ No effect	↔ No effect	◊ Mixed	Coach 52.5% vs. Athlete 87.8%; MS < HS (*p* = 0.001)
McKay et al. (51)	↑ Positive	↑ Positive	↔ No effect	↔ No effect	◊ Mixed	73.5% → 81.3% → 85.6% by strategy; no injury data
Suits et al. (55)	↑ Positive	↑ Positive	↔ No effect	↑ Positive	↑ Positive	KTA > Handout (*p* < 0.001); HR = 0.15 (*p* = 0.019)
Schoeb et al. (52)	↑ Positive	↑ Positive	↔ No effect	↑ Positive	↑ Positive	80% frequency; RR = 0.665 traumatic injuries
Lindblom et al. (50)	↑ Positive	↑ Positive	↔ No effect	↔ No effect	↑ Positive	74% adoption; 68–82% maintenance at 3 years
Ferri-Caruana et al. (46)	↑ Positive	↔ No effect	↑ Positive	↔ No effect	↑ Positive	89.7% compliance; FPPA −7.1° to −8.01°
Bulow et al. (45)	↑ Positive	↔ No effect	↔ No effect	↔ No effect	◊ Mixed	100% compliance; no significant improvements

Direction classification criteria: Positive, statistically significant improvement (*p* < 0.05) or ≥10% absolute difference favouring intervention; Negative, significant worsening or ≥10% difference favouring control; No effect, non-significant with <10% difference; Mixed, conflicting directions across outcomes within domain; NR, not reported.

**Table 6:** Effect direction vote count summary.

Outcome domain	↑ Positive	↓ Negative	↔ No effect	◊ Mixed	Direction consistency
Implementation compliance	12/13 (92%)	0/13 (0%)	0/13 (0%)	1/13 (8%)	Consistent positive
Adoption/reach	5/13 (38%)	0/13 (0%)	8/13 (62%)	0/13 (0%)	Limited reporting
Biomechanical outcomes	6/13 (46%)	0/13 (0%)	7/13 (54%)	0/13 (0%)	Positive when measured
Injury prevention	6/13 (46%)	0/13 (0%)	7/13 (54%)	0/13 (0%)	Positive when measured
Overall study direction	10/13 (77%)	0/13 (0%)	0/13 (0%)	3/13 (23%)	Predominantly positive

Implementation compliance outcomes showed positive directions in 12 of 13 studies (92%) reporting favourable results. No studies reported negative compliance directions (Table 6). One study (54) showed mixed compliance outcomes due to differential coach and athlete responses (52.5% vs. 87.8% respectively). Adoption and reach outcomes were reported in 5 of 13 studies (38%), with positive directions observed when measured. Eight studies (62%) did not report these outcomes (Table 6).

Effectiveness outcomes showed positive directions when measured. Five studies evaluated biomechanical outcomes (46, 47, 49, 53, 57), all demonstrating positive directions with moderate to large effect sizes (Table 5). Five studies reported injury outcomes (1–5), all showing positive directions with risk reductions ranging from 33.5 to 85%. Importantly, none of the studies showed negative effect directions across any outcome domain, indicating that neuromuscular training programs are not harmful when conducted.

Vote counting synthesis organized by implementation strategy category showed differential patterns across strategy types (Table 7). Educational institution settings with professional supervision (51, 56, 57) showed consistent positive directions for compliance outcomes (3/3 studies) and injury outcomes (2/3 studies), with mean compliance rates of 96.1%. Technology-enhanced methods exhibited universally positive compliance directions with limited injury outcome data. School-based interventions with fluctuating professional assistance showed uneven trends, supporting the importance of continuous supervision. Self-directed and community-based strategies yielded positive paths when there was sufficient stakeholder involvement or motivation among the participants.

**Table 7:** Effect direction by implementation strategy category.

Implementation strategy	Studies (n)	Compliance direction	Injury direction	Overall	Evidence summary
Professional supervision + educational institution	3	↑ Positive	↑ Positive	↑ Positive	96.1% mean compliance; 2/3 injury reduction
Technology-enhanced research supervision	2	↑ Positive	↔ No effect	↑ Positive	93.7% mean compliance; biomechanical only
School-based with professional support	2	◊ Mixed	↑ Positive	◊ Mixed	>95% vs. 52.5% compliance; variable by support
Community-based with variable support	2	↑ Positive	↑ Positive	↑ Positive	Strategy-dependent; KTA superior to handout
Self-directed/minimal supervision	2	↑ Positive	↑ Positive	↑ Positive	77% mean compliance; sustainable long-term
Research laboratory controlled	2	↑ Positive	↔ No effect	◊ Mixed	94.9% compliance; effectiveness variable


Studies grouped by primary implementation strategy to examine strategy-outcome relationships. Direction reflects predominant pattern within each strategy category.

The systematic investigation of the sources of heterogeneity revealed multiple factors that explain the variation in the directions of the effect (Table 8). The level of professional support proved to be the most powerful distinguishing factor, as high-support studies obtained 12/13 positive compliance directions in contrast to mixed outcomes in minimal-support settings. The implementation frequency exhibited a threshold effect that aligned with the dose–response relationship: the positive direction of injury prevention was consistently positive in studies with 2 or more sessions per week (6/6), whereas studies with fewer sessions/week reported no positive effects of injury prevention (0/3). Setting type also affected consistency, with educational settings reporting more consistent positive directions (5/5) than community settings (3/4). The MMAT quality of study indicated consistent direction patterns across high-quality (7/8 positive) and moderate-quality studies (3/5 positive or mixed), implying that approach rigor did not substantially impact effect direction.

**Table 8:** Sources of heterogeneity in effect directions.

Heterogeneity source	Categories compared	Effect pattern	Interpretation
Professional support level	High vs. Minimal	High: 12/13 positive compliance; Minimal: 1/2 mixed	Professional support consistently associated with positive implementation outcomes
Setting type	Educational vs. Community	Educational: 5/5 positive overall; Community: 3/4 positive	Educational settings show more consistent positive directions
Implementation frequency	≥2x/week vs. <2x/week	≥2x/week: 6/6 positive injury; <2x/week: 0/3 positive	Frequency threshold critical for injury prevention effectiveness
Study quality	High vs. Moderate MMAT	High: 7/8 positive; Moderate: 3/5 positive or mixed	Higher quality studies show similar direction patterns
Outcome measurement	Injury vs. Biomechanical	Injury: 6/6 positive when measured; Biomech: 6/6 positive when measured	Both outcome types show consistent positive when adequately measured
Follow-up duration	≤12 weeks vs. >12 weeks	Short: 8/9 positive; Long: 4/4 positive	Positive directions maintained across follow-up durations

These findings must be interpreted acknowledging vote counting limitations. This method assigns equal weight regardless of sample size, cannot detect small effects, and may oversimplify complex patterns. The absence of standardized effect sizes precluded meta-analytic pooling; therefore, effect directions indicate outcome pattern consistency rather than precise magnitude estimates. No studies reported negative effect directions across implementation or effectiveness outcome domains, though this pattern should be interpreted cautiously given potential publication bias and the limitations of vote counting methodology.

### 3.7 Sensitivity analysis

Sensitivity analyses were conducted to assess the robustness of synthesis findings to study quality variations and effect direction classification decisions.

#### 3.7.1 Study quality sensitivity

To assess whether methodological quality influenced synthesis conclusions, analyses were repeated restricting inclusion to high-quality studies only [*n* = 8; MMAT rating ‘High’: Bulow et al. (45), Ford et al. (47), Foss et al. (48), Hopper et al. (49), Shams et al. (53), Suits et al. (55), Yarsiasat et al. (56), and Zebis et al. (57)] compared with moderate-quality studies [n = 5: Ferri-Caruana et al. (46), Lindblom et al. (50), McKay et al. (51), Schoeb et al. (52), and Sugimoto et al. (54)]. Limiting to high-quality studies did not change the direction or consistency of primary findings (Table 9). High-quality studies showed consistently positive implementation compliance outcomes (8/8, 100%) compared to moderate-quality studies (4/5, 80%), with the one mixed finding explained by the coach-athlete compliance difference reported by (54). The implementation strategy hierarchy was maintained at the levels of quality, where professional supervision models had a higher compliance rate (range: 89–100) than educational-only models irrespective of the study quality classification.

**Table 9:** Effect direction patterns by study quality.

Outcome domain	High quality (*n* = 8)	Moderate quality (*n* = 5)	Interpretation
Implementation compliance: positive	8/8 (100%)	4/5 (80%)	Consistent across quality
Adoption/reach: positive	3/8 (38%)	2/5 (40%)	Similar reporting gaps
Biomechanical outcomes: positive	4/8 (50%)	2/5 (40%)	Consistent when measured
Injury prevention: positive	4/8 (50%)	2/5 (40%)	Consistent when measured
Overall study direction: positive	7/8 (88%)	3/5 (60%)	Similar overall pattern
Professional support advantage confirmed	Yes	Yes	Robust finding
Frequency threshold (≥2x/week) supported	Yes (55)	Not directly tested	High-quality evidence

The positive directions in injury prevention outcomes were observed in high-quality studies reporting injury outcomes included (48, 55–57) and moderate-quality studies included (52, 54). The finding that implementation ≥2 sessions per week was associated with 85% ACL injury risk reduction was from one high-quality study (55): HR = 0.15, 95% CI = 0.03–0.73, *p* = 0.019 (Table 10). Likewise, high-quality evidence by Foss et al. (48) (injury rate ratio: 5.34 vs. 8.54/1000 athlete-exposures, *p* < 0.001) and Zebis et al. (57) (1 vs. 7 lower extremity injuries, *p* = 0.04) supported the dose–response relationship between implementation quality and effectiveness. The coach implementation pattern, where athlete compliance (87.8%) exceeded coach implementation (52.5%), was observed in one moderate-quality study (54) (Table 9). Similar patterns of higher implementation with professional support were observed across both high-quality and moderate-quality studies, suggesting this pattern was not limited to one quality stratum.

**Table 10:** Implementation success factors and evidence-based thresholds.

Implementation success factor	Evidence source(s)	Threshold/benchmark identified	Implementation strategy implications
Implementation frequency			
Minimum effective dose	Suits et al. (55)	≥2 sessions per week for 85% ACL injury risk reduction (HR = 0.15, *p* = 0.019)	All strategies must achieve minimum frequency threshold
Seasonal consistency	Schoeb et al. (52)	80% of recommended frequency (0.8 ± 0.6/week) still effective for injury reduction	Perfect adherence not required for effectiveness
Professional support level			
Ongoing supervision requirement	Sugimoto et al. (54) vs. Foss et al. (48)	Coach compliance: 52.5% (minimal support) vs. > 95% (AT oversight)	Professional support essential for coach-led implementation
Monitoring frequency optimization	Zebis et al. (57)	Bi-weekly research team visits achieved 89% compliance	Regular but not daily monitoring sufficient
Stakeholder engagement			
Active vs. passive strategies	Suits et al. (55); McKay et al. (51)	KTA intervention > educational handout (*p* < 0.001); Comprehensive > workshop > control	Stakeholder engagement improves implementation rates
Barrier identification systems	Suits et al. (55)	Systematic barrier assessment and adaptation increased the implementation	Proactive barrier management essential
Educational institution advantages			
Implementation environment	Zebis et al. (57); Yarsiasat et al. (56)	89–100% completion in educational settings	Educational institutions facilitate high-fidelity implementation
Teacher training models	Zebis et al. (57)	2-h seminar + ongoing support achieved high compliance	Comprehensive training investment required
Technology integration			
Enhancement effectiveness	Ford et al. (47); Shams et al. (53)	Technology-enhanced approaches achieved 93–94% completion	Technology can improve implementation but increases resource requirements
Scalability limitations	Ford et al. (47)	High-tech approaches limit widespread implementation potential	Balance enhancement benefits with scalability needs
Long-term Sustainability			
Maintenance rates	Lindblom et al. (50)	68–82% continued use at 3-year follow-up	Programs can be sustained with appropriate organizational support
Fidelity degradation	Lindblom et al. (50)	Only 23–26% used programs without modifications	Implementation fidelity challenges over time
Population-specific factors			
Age group considerations	Sugimoto et al. (54)	Middle school coaches less compliant than high school (*p* = 0.001)	Age-specific implementation strategies needed
Elite vs. amateur populations	Schoeb et al. (52) vs. Suits et al. (55)	Elite athletes more self-directed; amateur athletes require more support	Population characteristics influence optimal implementation approach
Implementation quality vs. Strategy			
Primary determinant	Suits et al. (55)	Implementation frequency more important than strategy choice	Focus resources on achieving implementation quality over strategy selection
Coach vs. athlete readiness	Sugimoto et al. (54)	Athletes ready (87.8% compliance) when coaches implement (52.5% compliance)	Coach implementation capacity is the primary bottleneck.

#### 3.7.2 Effect direction classification sensitivity

The strength of effect direction categories was also assessed with alternative thresholds. The main analysis grouped positive directions by statistical significance (*p* < 0.05) or by clinically meaningful differences (at least 10 percent absolute difference in compliance, at least 20 percent decrease in relative risk of injury). Stricter thresholds (*p* < 0.01 or ≥15% absolute difference) reduced positive classifications to 77% (10/13 studies) but maintained the pattern of no negative effect directions and similar strategy-level patterns (Tables 6, 11). A higher liberal threshold (*p* < 0.10 or 5% or greater absolute difference) raised positive classifications to 85% (11/13) without changing main conclusions. No negative effect directions were observed under any threshold specification. This pattern was consistent across threshold levels, though interpretation should account for potential publication bias toward positive findings. Sensitivity analyses showed that main synthesis patterns were consistent across study quality strata and effect direction threshold specifications. Key patterns including associations between implementation quality and outcomes, professional support and higher compliance rates, and the implementation frequency-effectiveness relationship observed in one study (55), remained consistent across analytical approaches.

**Table 11:** Implementation barriers and facilitators by CFIR domain.

CFIR domain	Primary barriers	Primary facilitators	Frequency across studies	Representative studies
Intervention characteristics				
Complexity	Time burden (15–30 min sessions); multi-component learning requirements	Program simplicity; Clear progression protocols; Evidence-based design	Barriers: 8/13 studies; Facilitators: 11/13 studies	Sugimoto et al. (54); Zebis et al. (57)
Adaptability	Limited modification options; Rigid protocols	Flexible delivery options; Sport-specific adaptations; RPE-guided progression	Barriers: 4/13 studies; Facilitators: 9/13 studies	Schoeb et al. (52); Yarsiasat et al. (56)
Relative advantage	Limited immediate observable benefits; Performance vs. prevention focus	Injury prevention + performance enhancement; Strong evidence base	Barriers: 5/13 studies; Facilitators: 10/13 studies	McKay et al. (51); Lindblom et al. (50)
Outer setting				
External policies	Competition scheduling pressure; Lack of district-level policies	National FA support; Organizational partnerships; Research credibility	Barriers: 4/13 studies; Facilitators: 8/13 studies	Sugimoto et al. (54); Lindblom et al. (50)
Competitive pressure	Performance priorities vs. injury prevention time	Integration with existing training; Organizational endorsement	Barriers: 6/13 studies; Facilitators: 5/13 studies	Schoeb et al. (52); Foss et al. (48)
Inner setting				
Available resources	Limited equipment; Inadequate facilities; No organizational routines	Existing AT infrastructure; University resources; Equipment provision	Barriers: 5/13 studies; Facilitators: 12/13 studies	Lindblom et al. (50); Ford et al. (47)
Leadership engagement	Variable coach commitment; Absent organizational support	Strong institutional support; Teacher/coach training; Research partnerships	Barriers: 7/13 studies; Facilitators: 10/13 studies	Sugimoto et al. (54); Zebis et al. (57)
Individual characteristics				
Knowledge and beliefs	Coach implementation burden; Knowledge gaps about injury prevention	High athlete receptivity; Professional expertise; Evidence awareness	Barriers: 8/13 studies; Facilitators: 9/13 studies	McKay et al. (51); Foss et al. (48)
Self-efficacy	Coach confidence limitations; Implementation skill gaps	Professional qualifications; Experience levels; Training provision	Barriers: 6/13 studies; Facilitators: 8/13 studies	Sugimoto et al. (54); Hopper et al. (49)
Implementation process				
Engaging	Minimal ongoing support; Limited stakeholder involvement	Comprehensive training; Regular monitoring; Stakeholder consultation	Barriers: 7/13 studies; Facilitators: 11/13 studies	Sugimoto et al. (54); Suits et al. (55)
Executing	No systematic monitoring; Quality control absence	Professional oversight; Regular supervision; Quality assurance	Barriers: 5/13 studies; Facilitators: 12/13 studies	Lindblom et al. (50); Zebis et al. (57)

### 3.8 Barriers and facilitators analysis

The Consolidated Framework for Implementation Research (CFIR) analysis revealed systematic patterns of barriers and facilitators across the five implementation domains, with intervention characteristics and implementation process factors emerging as the most critical determinants of implementation success (Table 11).

#### 3.8.1 Program complexity

Program complexity represented the most frequently cited barrier across eight studies (45, 47, 48, 51–55), with coaches consistently reporting that 15–30 min session requirements and multi-component learning demands created significant implementation burdens. Conversely, program simplicity and clear progression protocols served as powerful facilitators in eleven studies (46–53, 55–57), with structured approaches like the evidence-based neuromuscular training program achieving 89% compliance through systematic progression and regular monitoring. The complexity-simplicity paradox demonstrates that while comprehensive programs may be more effective, implementation success depends critically on presenting complex interventions through simplified, manageable delivery protocols.

#### 3.8.2 Intervention adaptability

Intervention adaptability emerged as a key implementation determinant, with rigid protocols creating barriers in four studies (45, 51, 53, 54) while flexible delivery options facilitated implementation in nine studies (46–49, 51–53, 55, 56). The self-directed ISPAInt program exemplified successful adaptability by allowing athletes to perform exercises “anytime and anywhere within 20 minutes,” achieving 80% of the recommended frequency despite minimal supervision (52). Sport-specific adaptations proved particularly valuable, with the PEP program’s successful modification for Sepak Takraw demonstrating that cultural and sport-specific customization enhances acceptability without compromising effectiveness (56). This finding underscores that implementation frameworks must balance standardization with contextual adaptation to maximize real-world adoption.

#### 3.8.3 External setting factors

External setting factors revealed substantial organizational influence on implementation success, with supportive policies facilitating implementation in eight studies (47–54, 57) while competitive pressures created barriers in six studies (48, 51, 52, 54, 55, 57). National-level policy support provided crucial implementation infrastructure, as demonstrated by the Swedish Football Association’s formal policies enabling widespread program reach (91–99% familiarity) three years post-trial (50). However, the absence of district-level implementation policies created significant gaps, with 0 % of district associations maintaining formal guidelines despite national support. The tension between performance priorities and injury prevention time allocation consistently emerged as a barrier, particularly in competitive environments where coaches faced pressure to maximize skill development over prevention activities (52, 54).

#### 3.8.4 Inner setting factors

Inner setting characteristics highlighted the critical importance of organizational resources and leadership engagement for implementation success. Available resources served as facilitators in twelve of thirteen studies (45–53, 55–57), with existing athletic trainer infrastructure proving particularly valuable for school-based implementations. University research partnerships provided essential equipment and expertise, enabling high-fidelity implementation in controlled settings (47, 49). Leadership engagement emerged as highly variable, with strong institutional support facilitating implementation in ten studies (45, 47–52, 54, 55, 57) while absent organizational commitment created barriers in seven studies (45, 50–55). The stark contrast between the >95% team compliance achieved with athletic trainer oversight compared to 52.5% coach compliance with minimal support illustrates the transformative impact of organizational leadership and professional support systems.

#### 3.8.5 Individual factors

Individual characteristics revealed a consistent pattern where knowledge gaps and implementation burdens created coach-level barriers while athlete receptivity remained consistently high. Coach implementation challenges appeared in eight studies (45, 48, 50–55), with knowledge gaps about injury prevention mechanisms and implementation skill deficits limiting program delivery. Paradoxically, athletes demonstrated high compliance (87.8%) when programs were delivered, indicating that implementation failure occurred primarily at the provider rather than recipient level (54). Professional qualifications and comprehensive training served as powerful facilitators, with accredited strength and conditioning coaches achieving 100% completion rates through technical competency and safety prioritization (49). This finding suggests that implementation strategies must focus primarily on enabling and supporting coaches rather than motivating athletes.

#### 3.8.6 Implementation process

Implementation process factors showed associations between ongoing engagement and implementation success. Minimal ongoing support created barriers in seven studies (45, 50–55), while comprehensive training and regular monitoring facilitated implementation in eleven studies (45–51, 53, 55–57). The Knowledge-to-Action framework’s success in achieving significantly higher implementation rates compared to educational handouts exemplifies the value of systematic stakeholder engagement and barrier identification (55). Professional oversight emerged as essential for execution quality, with systematic monitoring enabling successful implementation across twelve studies while its absence led to implementation failures (45–53, 55–57). Patterns across the implementation process domain showed that sustained, systematic support was associated with higher implementation outcomes compared to one-time training or passive information delivery.

### 3.9 Real-world effectiveness evidence

Three studies provided ACL-specific injury incidence data with quantifiable implementation contexts. Suits et al. (55) reported that among amateur female soccer players, implementation ≥2 times per week was associated with 85% lower ACL injury risk compared to less frequent implementation (HR = 0.15, 95% CI = 0.03–0.73, *p* = 0.019). Yarsiasat et al. (56) reported reduced knee injury rates in the intervention group (11.5%) compared to control (34.6%) in female Sepak takraw players, including one partial versus three complete ACL tears. Zebis et al. (57) reported lower extremity injury reduction (1 vs. 7 injuries, *p* = 0.04) in female athletes at a sports college, though ACL-specific injuries were not separately reported. Three additional studies reported non-ACL-specific lower extremity or knee injury outcomes. Foss et al. (48) demonstrated overall injury rate reduction (5.34 vs. 8.54 per 1,000 athlete-exposures, p < 0.001; 1.6-fold reduction) across multiple sports. Schoeb et al. (52) reported traumatic injury reduction of 33.5% (RR = 0.665) and overuse injury reduction of 30.1% in youth skiers. Sugimoto et al. (5) reported implementation compliance data but did not report injury outcomes.

#### 3.9.1 Implementation-effectiveness relationship

Real-world effectiveness outcomes varied by implementation quality and context. Among the three studies reporting ACL-specific data, injury reductions ranged from 65 to 85% when programs were implemented with adequate frequency and supervision (Table 12). Suits et al. (55) found that implementation frequency ≥2 sessions per week was associated with 85% ACL injury risk reduction (HR = 0.15, 95% CI = 0.03–0.73, *p* = 0.019), providing direct dose–response evidence among included studies for implementation frequency and injury prevention effectiveness in amateur soccer settings. These findings indicate that meaningful injury reductions were achieved when implementation thresholds were met in the contexts studied.

**Table 12:** Implementation strategy effectiveness and real-world outcomes.

Implementation strategy category	Studies (n)	Mean compliance rate	Injury prevention effectiveness	Scalability assessment	Sustainability evidence
Professional supervision + Educational institution	3 Zebis et al. (57); Yarsiasat et al. (56); Hopper et al. (49)	96.1% (range: 89–100%)	High: Significant injury reductions and/or biomechanical improvements	Moderate: Requires educational infrastructure	Limited assessment
			Zebis: 1 vs. 7 injuries (*p* = 0.04); Yarsiasat: 11.5% vs. 34.6% knee injuries	Dependent on teacher/coach training systems	Only Zebis assessed beyond intervention
Technology-enhanced research supervision	2 Ford et al. (47); Shams et al. (53)	93.7% (range: 93.3–94.1%)	Moderate to high: Biomechanical improvements demonstrated	Low: High resource requirements	Not assessed
			Ford: All groups improved DVJ (*p* < 0.05); Shams: >50% LESS improvement	Requires specialized equipment and expertise	Research-only implementations
School-based with professional support	2 Foss et al. (48); Sugimoto et al. (54)	>75% (highly variable)	High: Demonstrated injury reduction	High: Leverages existing infrastructure	Limited assessment
			Foss: 1.6x injury reduction (*p* < 0.001)	AT infrastructure enables scaling	Not evaluated beyond the study period
Community-based with variable support	2 McKay et al. (51); Suits et al. (55)	73.5–85.6% (strategy-dependent)	Moderate: Strategy-dependent effectiveness	High: Community club systems	Long-term evidence mixed
			Suits: ≥2x/week implementation → 85% ACL risk reduction	Requires ongoing support systems	Suits: single season only
Self-directed/minimal supervision	2 Schoeb et al. (52); Lindblom et al. (50)	77% (range: 74–80%)	Moderate: Effective with motivated populations	Very High: Minimal resource requirements	Good evidence for sustainability
			Schoeb: 33.5% traumatic, 30.1% overuse injury reduction	Simple implementation model	Lindblom: 68–82% continued use at 3 years
Research laboratory controlled	2 Ferri-Caruana et al. (46); Bulow et al. (45)	94.9% (range: 89.7–100%)	Variable: Dependent on intervention appropriateness	Low: Research-specific conditions	Not applicable
			Ferri-Caruana: Significant FPPA improvements; Bulow: No benefits despite perfect implementation	High supervision requirements	Research implementations only

#### 3.9.2 Real-world vs. controlled setting effectiveness

Implementation fidelity emerged as a critical determinant of real-world effectiveness, with substantial variation observed between controlled and naturalistic settings across the included studies. Seven studies (45–47, 49, 53, 56, 57) achieved high implementation fidelity (>90% compliance) through intensive supervision models, while real-world implementations with minimal supervision demonstrated considerably lower adherence rates. Compliance rates varied substantially: Sugimoto et al. (5) reported 52.5% coach compliance with minimal support, while (48) achieved >95% team compliance with athletic trainer oversight (Table 12). Schoeb et al. (52) reported meaningful injury reductions (33.5% traumatic, 30.1% overuse) with 80% of recommended frequency in elite youth skiers, indicating that injury prevention benefits occurred despite suboptimal adherence in this motivated population.

The relationship between implementation strategies and real-world effectiveness revealed significant disparities in outcome achievement across different support models and settings. Educational institution-based implementations showed high effectiveness outcomes when measured, Zebis et al. (57) demonstrated significant reductions in lower extremity injuries (1 versus 7 injuries, *p* = 0.04) and Yarsiasat et al. (56) showing substantial decreases in knee injury incidence (11.5% versus 34.6%) when programs were delivered with professional supervision and organizational support (Table 12). Community-based implementation outcomes varied by strategy type, McKay et al. (51) demonstrated adherence rates ranging from 73.5 to 85.6% depending on support intensity, while Suits et al. (55) confirmed that active stakeholder engagement significantly improved implementation rates compared to passive information delivery. Self-directed implementations, exemplified by Schoeb et al. (52), achieved moderate effectiveness despite minimal supervision, but this success appeared contingent on elite athlete populations with high intrinsic motivation and organizational support structures.

#### 3.9.3 Implementation of quality thresholds for effectiveness

Perfect implementation fidelity did not ensure intervention effectiveness across all studies. Bulow et al. (45) achieved 100% implementation compliance but demonstrated no measurable benefits in dynamic balance outcomes, while (49) achieved similar fidelity (100% completion) with significant biomechanical improvements (knee abduction reduction *p* = 0.004, g = 1.43) (Table 12). These contrasting findings indicate that implementation fidelity alone does not determine effectiveness, and intervention appropriateness for the target population appears relevant. Across included studies, effectiveness outcomes varied by implementation quality, intervention characteristics, and population factors.

One study provided long-term sustainability evidence. Lindblom et al. (50) reported that 68–82% of original trial coaches continued program use three years post-trial, though only 23–26% maintained programs without modifications (Table 12). Program use continued in many coaches at three-year follow-up, though modifications to original programs were common. Real-world effectiveness outcomes were observed across multiple settings when adequate implementation support was provided, though long-term fidelity and sustainability data were limited (*n* = 1 study with ≥3-year follow-up).

## 4 Discussion

This systematic review addressed the implementation gap between the proven efficacy of neuromuscular training programs and their real-world effectiveness for ACL injury prevention in female athletes. The synthesis of 13 studies demonstrated consistent associations between implementation quality and program effectiveness, with substantial variations in adherence rates, implementation strategies, and contextual factors influencing outcomes. These findings suggest that evidence-based interventions require systematic implementation science approaches to achieve their potential effectiveness in practice settings, as intervention content alone does not ensure real-world impact.

### 4.1 Central role of implementation quality in program effectiveness

A key pattern emerging from this synthesis was the consistent association between implementation quality and program effectiveness, with implementation quality appearing to be an important determinant of real-world outcomes. Evidence from one high-quality study suggests that implementation frequency ≥2 times per week was associated with 85% lower ACL injury risk (HR = 0.15, 95% CI = 0.03–0.73, *p* = 0.019) among amateur female soccer players. In the same study, evidence was insufficient to establish differential injury effects across implementation strategy types, with one comparison finding no significant association between strategy choice and injury outcomes, though limited statistical power and heterogeneity preclude definitive conclusions. This pattern aligns with implementation science theory suggesting that the “voltage drop” between efficacy and effectiveness research is attributable to implementation factors in addition to intervention characteristics (26, 31, 58). The ≥2 sessions per week finding, while derived from a single study, provides a hypothesis-generating benchmark for effective implementation that warrants validation across diverse settings and populations. This evidence suggests the importance of reframing injury prevention research to address both “which programs work” and “how can programs be implemented effectively,” potentially influencing research priorities and resource allocation in sports medicine.

#### 4.1.1 Multi-level implementation barriers and the coach implementation paradox

Implementation patterns across multiple organizational levels showed differential compliance between implementers and participants in some settings. The findings showed that athletes achieved 87.8% compliance when programs were delivered, compared to only 52.5% coach compliance (54), indicating that implementation failure occurs at the provider rather than recipient level. This coach implementation paradox was further supported by evidence that high levels of coach intent to integrate prevention programs did not translate to effective implementation (16), suggesting that motivation alone is insufficient for successful adoption. The identification of this bottleneck aligns with the updated Consolidated Framework for Implementation Research (24), which emphasizes the critical role of implementation climate and individual characteristics in determining intervention success. These findings challenge traditional educational approaches to implementation and highlight the need for comprehensive coach support systems that address capacity, motivation, and opportunity barriers simultaneously.

#### 4.1.2 Implementation strategy effectiveness hierarchy

Implementation outcomes demonstrated patterns suggesting differential effectiveness across strategy types, with stakeholder-engaged professional support models associated with higher compliance rates than passive educational approaches. Studies employing comprehensive implementation support, including professional supervision (48), knowledge-to-action frameworks (55), and educational institution integration (56, 57), demonstrated superior implementation outcomes compared to educational-only strategies. This hierarchy aligns with implementation science evidence suggesting that active implementation strategies are more effective than passive dissemination approaches (24, 25). The success of professional support models validates the National Athletic Trainers’ Association position statement recommendations for multi-stakeholder involvement in prevention program implementation (59). However, the resource intensity of effective implementation strategies creates tension between implementation effectiveness and scalability, necessitating careful consideration of cost-effectiveness in implementation planning.

#### 4.1.3 Contextual factors and setting-specific implementation patterns

The synthesis revealed contextual variation in implementation outcomes, with educational institutions demonstrating higher implementation fidelity compared to community and elite sports settings in the studies reviewed. Studies conducted in school-based settings (48) and sports colleges (57) achieved exceptional compliance rates and injury reduction outcomes, suggesting that structured educational environments provide optimal implementation contexts. This pattern may reflect the inherent characteristics of educational settings, including dedicated time allocation, professional supervision, and organizational commitment to holistic athlete development. Conversely, community-based implementations showed greater variability, with success dependent on comprehensive support provision and stakeholder engagement intensity. Elite sports settings presented unique challenges, with high athlete motivation potentially offset by competing performance priorities and time constraints. These contextual patterns align with implementation science literature emphasizing the importance of inner-setting characteristics in determining intervention success (24, 25, 28, 31).

### 4.2 Theoretical framework integration and mechanistic understanding

The application of implementation science frameworks, particularly the Consolidated Framework for Implementation Research and the RE-AIM model, provided a systematic understanding of implementation mechanisms and outcomes. CFIR-based analysis revealed that intervention characteristics, while important, were less influential than individual characteristics and implementation process factors in determining success. The identification of implementation process factors as critical determinants supports the argument that successful ACL prevention requires co-creation approaches involving context appreciation and stakeholder engagement (60, 61). RE-AIM framework application demonstrated that while most programs achieved adequate reach and adoption, implementation fidelity and maintenance remained significant challenges. This pattern aligns with observations that long-term sustainability represents a critical gap in current prevention efforts (13, 62). The theoretical framework integration provides a foundation for developing evidence-based implementation strategies that address multiple determinants simultaneously.

#### 4.2.1 Technology integration and motor learning considerations

Emerging evidence from this review suggested that technology-enhanced implementation approaches may improve biomechanical outcomes, though with scalability considerations. Our results demonstrated that real-time biofeedback significantly improved biomechanical outcomes (47), while simple augmentation strategies such as mirror feedback and taping could enhance traditional training approaches (53). These findings align with neurocognitive and ecological motor learning frameworks, which emphasize the importance of feedback modalities in optimizing skill acquisition and retention (61). However, technology-enhanced approaches often require specialized equipment and training, creating potential barriers to widespread implementation. The integration of motor learning principles into implementation strategies represents an evolving area where sports science and implementation science intersect, offering opportunities for innovation in delivery methods while requiring careful consideration of implementation feasibility.

#### 4.2.2 Implementation-effectiveness relationships and dose–response evidence

Patterns across included studies suggested relationships between implementation quality and effectiveness outcomes. The ≥2 sessions per week finding represents a minimum dose threshold rather than evidence of a graded dose–response relationship, as few studies tested multiple frequency levels systematically. True dose–response relationships, where effectiveness increases proportionally across multiple dose levels, require investigation in future research (45, 57). High-fidelity implementation resulted in significant neuromuscular adaptations associated with ACL injury risk reduction (57), while perfect implementation of inappropriate interventions yielded no benefits (45). These findings establish implementation fidelity as a critical mediator in the relationship between intervention exposure and effectiveness outcomes. The dose–response evidence provides practitioners with specific targets for implementation success while highlighting the importance of matching intervention appropriateness to target populations. This relationship validates meta-analytic evidence suggesting that compliance represents a key moderator of intervention effectiveness (5, 16).

#### 4.2.3 Stakeholder engagement and co-creation approaches

The review provided strong evidence supporting stakeholder engagement and co-creation approaches in implementation strategy development. Studies employing systematic barrier identification and adaptation strategies (55) achieved superior implementation outcomes compared to standardized approaches. This finding validates the Knowledge-to-Action framework’s emphasis on tailored implementation strategies based on local context and stakeholder needs. The success of stakeholder engagement approaches aligns with implementation science literature demonstrating that participatory implementation strategies improve adoption and sustainability (24, 31, 58). However, the resource intensity of comprehensive stakeholder engagement creates practical challenges for widespread implementation, necessitating the development of scalable engagement approaches that maintain effectiveness while reducing resource requirements.

### 4.3 The knowledge-practice translation challenge

The findings revealed persistent challenges in translating implementation knowledge into practice, despite growing awareness of implementation barriers. Educational interventions alone, while improving coach knowledge and attitudes (7), failed to achieve sustained implementation improvements (16). This pattern highlights the complexity of behaviour change in sports settings and the need for multi-component implementation strategies that address cognitive, motivational, and environmental factors simultaneously. The seven-step implementation framework (3) provides a systematic approach to addressing these challenges, emphasizing the importance of organizational support, stakeholder engagement, and ongoing evaluation. However, the gap between knowledge and practice persists, suggesting that the implementation science principles must be more systematically integrated into sports medicine education and practice.

### 4.4 Long-term sustainability and maintenance challenges

The synthesis revealed concerning patterns regarding long-term sustainability and program maintenance across included studies. While most programs achieved initial adoption and short-term implementation success, evidence for sustained implementation beyond research periods remained limited. The three-year follow-up findings (50) demonstrated that despite initial success, implementation fidelity decreased over time, with only 23–26% of coaches using programs without modifications. This pattern aligns with the implementation science understanding that maintenance represents the most challenging phase of implementation (24). The lack of systematic sustainability planning in most studies highlights a critical gap in current implementation approaches and emphasizes the need for explicit attention to maintenance factors in future implementation research.

### 4.5 Cultural and contextual adaptation considerations

The review identified successful implementation across diverse cultural contexts, from Danish sports colleges (57) to Thai sports schools (56) to American school districts (48), suggesting that evidence-based programs can be effectively adapted while maintaining core intervention components. However, the specific adaptations required for different contexts remained underexplored in most studies. The success of culturally adapted programs (56) supports the importance of surface-level adaptations while preserving deep structural elements, aligning with cultural adaptation frameworks in implementation science. These findings emphasize the need for systematic approaches to cultural adaptation that balance intervention fidelity with contextual appropriateness.

### 4.6 Practical implications and evidence-based recommendations

The findings of this review provide several actionable recommendations for practitioners and organizations seeking to implement neuromuscular training programs effectively. Organizations should prioritize implementation support over program selection, ensuring adequate professional supervision and ongoing assistance for implementers. The establishment of ≥2 sessions per week as a minimum implementation threshold provides clear quality targets for program delivery. Educational institutions should be prioritized as early implementation sites due to their superior implementation environments. Comprehensive stakeholder engagement, while resource-intensive, should be considered essential for sustainable implementation success. Organizations should develop systematic approaches to barrier identification and adaptation, rather than relying on standardized implementation protocols. The integration of implementation science principles into sports medicine practice requires investment in professional development and organizational capacity building to support evidence-based implementation approaches. Finally, the narrative synthesis approach employed in this review (22) demonstrates the value of systematic non-meta-analytic synthesis for understanding complex implementation phenomena that cannot be adequately captured through traditional quantitative approaches.

### 4.7 Implementation science implications for sports medicine

This review establishes implementation science as an essential discipline for sports medicine, with implications extending beyond ACL injury prevention to broader sports injury prevention efforts. The identification of implementation quality as a primary determinant of effectiveness challenges traditional sports medicine research priorities and suggests that implementation research should receive equal attention to efficacy research. The development of evidence-based implementation thresholds provides a foundation for quality assurance in prevention program delivery. Furthermore, the recognition of complex multi-level implementation barriers necessitates interdisciplinary collaboration between sports medicine professionals, implementation scientists, and behavioural researchers. These findings support calls for the implementation of science training in sports medicine education and the development of implementation support systems within sports organizations.

### 4.8 Limitations

Several limitations must be acknowledged in interpreting these findings. The heterogeneity of implementation measures across studies precluded quantitative synthesis, necessitating narrative approaches that may be subject to interpretation bias despite systematic framework application. The predominance of short-term studies (6–12 weeks) restricted understanding of long-term sustainability patterns and maintenance challenges, with only one study providing extended follow-up data beyond the intervention period. The exclusive focus on female athletes, while addressing a critical epidemiological need, limits generalizability to male athletes and mixed-gender programs. Implementation outcome measurement varied considerably across studies, with some relying on self-reported adherence while others employed objective monitoring, potentially introducing measurement bias. The majority of included studies originated from high-income countries with established sports medicine infrastructure, limiting applicability to resource-constrained settings. Additionally, most studies focused on coach-led implementation with limited examination of alternative delivery models or stakeholder perspectives beyond coaches and athletes. Publication bias may have influenced results, as studies with positive implementation outcomes may be more likely to be published. Finally, the synthesis approach, while appropriate for the heterogeneous data, cannot establish causal relationships between specific implementation strategies and outcomes with the same precision as meta-analytic approaches. These limitations highlight the need for standardized implementation outcome measures and longitudinal research designs in future studies.

#### 4.8.1 Clinical threshold justification

Effect direction classification thresholds (*p* < 0.05 for statistical significance; ≥10% absolute difference for compliance/adherence outcomes; ≥20% relative risk reduction for injury outcomes) were defined *a priori* as pragmatic benchmarks representing clinically meaningful differences in implementation science contexts. The 10% absolute compliance difference threshold was selected based on reviewer judgment that this magnitude of difference is likely to translate into meaningful population-level impact when scaled across programs, though no universal consensus exists for implementation outcome thresholds. The 20% relative injury risk reduction threshold reflects a clinically important effect size in injury prevention research. These thresholds represent pragmatic decisions to enable systematic evidence synthesis, and sensitivity analyses examined whether conclusions changed under alternative specifications (Section 3.7).

#### 4.8.2 Publication bias and selective reporting

Publication bias should be considered when interpreting these findings. Studies demonstrating successful implementation and positive injury prevention outcomes may be more likely to achieve publication than those reporting implementation failures or null results, potentially overestimating the perceived effectiveness of neuromuscular training programs. The absence of negative effect directions across all 13 included studies, while encouraging regarding program safety, could partly reflect selective publication rather than universal effectiveness. Conventional statistical assessments of publication bias (funnel plot asymmetry, Egger regression tests) require pooled effect sizes and could not be applied to this narrative synthesis of heterogeneous implementation evidence.

Selective outcome reporting may also influence findings, as only 38% of studies (5/13) reported adoption and reach outcomes, 46% (6/13) reported injury prevention outcomes, and 23% (3/13) reported ACL-specific injury outcomes. This pattern could indicate selective reporting of positive outcomes rather than comprehensive outcome measurement. Pre-registration of protocols was not uniformly reported across included studies, restricting systematic assessment of reporting completeness. Future implementation research should prioritize prospective registration with full outcome specification to enhance transparency and minimize selective reporting concerns.

#### 4.8.3 Safety assessment limitations

The absence of reported negative effect directions should not be interpreted as definitive evidence of safety or absence of harm. Adverse events beyond participant withdrawals were not systematically assessed or reported across studies. No studies explicitly reported systematic adverse event monitoring protocols, limiting confidence in safety conclusions. The finding that no studies reported increased injury risk with neuromuscular training should be stated as “no included studies reported increased injury risk” rather than concluding that programs are universally safe, acknowledging underreporting limitations and the general inadequacy of included studies to detect rare adverse events.

## 5 Conclusion

This systematic review synthesizes available evidence on implementation science for neuromuscular training programs in ACL injury prevention, providing insights that highlight the importance of systematic implementation approaches in sports injury prevention research and practice. The finding that implementation quality appears to be a key correlate of program effectiveness, potentially as important as program selection in determining real-world outcomes, suggests the need to expand prevention strategies from content-focused to process-focused approaches. Evidence from one high-quality study suggests that implementation ≥2 sessions per week may represent an important threshold, associated with 85% ACL injury risk reduction (HR = 0.15, 95% CI = 0.03–0.73) though this hypothesis-generating finding requires validation across diverse settings, populations, and program types.

### 5.1 Implementation science contributions to sports medicine

In one school-based study with minimal professional support, athletes demonstrated 87.8% compliance when programs were delivered, while coaches achieved only 52.5% implementation rates, suggesting that in similar contexts, provider capacity may be a more critical barrier than participant motivation. This pattern indicates the importance of implementation support systems that extend beyond traditional educational approaches toward systematic capacity-building initiatives addressing motivational, capability, and opportunity barriers simultaneously. Educational institutions demonstrated consistently high implementation outcomes across three studies, suggesting these settings may be suitable venues for early adoption efforts and pilot program development.

### 5.2 Clinical and policy implications

Implementation outcomes demonstrated patterns consistent with an effectiveness gradient, wherein stakeholder-engaged professional support models were associated with higher compliance rates than passive dissemination approaches, though this pattern was observed primarily for implementation process outcomes rather than injury outcomes. Th resource intensity required for effective implementation creates practical tensions between effectiveness and scalability that must be addressed through innovative delivery models and systematic cost-effectiveness research. Sports organizations must develop implementation support infrastructure that provides ongoing assistance rather than one-time educational interventions, while healthcare systems should consider the integration of implementation support roles, particularly athletic trainers and physiotherapists, into systematic injury prevention efforts.

### 5.3 Theoretical framework validation and development

The successful application of implementation science frameworks, particularly CFIR and RE-AIM, to sports medicine research demonstrates the value of theory-driven approaches to understanding complex implementation phenomena. The synthesis revealed that barriers and facilitators most frequently mapped to individual characteristics and implementation process domains within the CFIR framework, suggesting these may be important leverage points for intervention, though causal relationships cannot be established from the available evidence. This finding is consistent with calls for interdisciplinary collaboration between sports medicine professionals, implementation scientists, and behavioural researchers in developing comprehensive prevention strategies. Future implementation efforts should systematically apply these frameworks to ensure comprehensive assessment of implementation determinants and outcomes.

### 5.4 Research gaps and future directions

Several critical evidence gaps emerged from this synthesis. The predominance of short-term studies (only 1 study with ≥3-year follow-up) severely limits understanding of sustainability and maintenance patterns. ACL-specific injury outcomes were reported in only 3 of 13 studies, with broader injury outcomes in 6 studies, limiting definitive conclusions about injury prevention effectiveness across settings. The lack of standardized implementation outcome measures across studies hampers comparative effectiveness research and meta-analytic synthesis possibilities. Additionally, limited examination of cost-effectiveness relationships between implementation strategies and injury prevention outcomes restricts evidence-based resource allocation decisions.

Future research priorities should include: (1) longitudinal designs with ≥2-year follow-up to assess maintenance and fidelity over time; (2) standardized measurement of ACL-specific injury outcomes with exposure adjustment across diverse settings; (3) developing scalable implementation interventions that maintain effectiveness while reducing resource requirements; and (4) investigating technology-enhanced implementation approaches, recognizing that while technology shows promise for biomechanical outcomes, its application to implementation success and injury prevention requires rigorous testing of underlying theoretical mechanisms rather than assumption of effectiveness.

Notably, ACL injury outcomes were reported in only 3 of 13 included studies, with broader injury outcomes in 6 studies. Adoption, reach, and maintenance outcomes were inconsistently measured, limiting definitive comparisons across implementation strategies and settings. These evidence gaps should be considered when interpreting recommendations and applying findings to specific contexts. Future research should prioritize standardized implementation outcome measurement and long-term sustainability assessment to strengthen the evidence base for implementation strategies in sports injury prevention.

## Data Availability

The original contributions presented in the study are included in the article/Supplementary material, further inquiries can be directed to the corresponding author.
