# Supplementary material for: Implementation effectiveness, barriers, and real-world outcomes of neuromuscular training programs for ACL injury prevention in female athletes: systematic review with narrative synthesis using SWiM framework
Source: Front Public Health. 2026 Feb 24;14:1743075. doi: 10.3389/fpubh.2026.1743075 (PMC12971891; doi:10.3389/fpubh.2026.1743075)
Supplement: Supplementary file 1 [file Supplementary_file_1.docx]

**Appendix 1: Database Search Strategies**

| **Database** | **Search Strategy** | **Results** |
| --- | --- | --- |
| PubMed | ((("anterior cruciate ligament"[MeSH Terms] OR "anterior cruciate ligament"[tiab] OR "ACL"[tiab] OR "knee injuries"[MeSH Terms]) AND ("Athletic Injuries/prevention and control"[MeSH Terms] OR "injury prevention"[tiab] OR "prevention"[tiab])) AND ("Exercise Therapy"[MeSH Terms] OR "neuromuscular training"[tiab] OR "neuromuscular exercise"[tiab] OR "injury prevention program"[tiab] OR "prevention program"[tiab] OR "FIFA 11+"[tiab] OR "PEP program"[tiab] OR "KLIP"[tiab] OR "Sportsmetrics"[tiab])) AND ("implementation"[tiab] OR "compliance"[tiab] OR "adherence"[tiab] OR "adoption"[tiab] OR "real world"[tiab] OR "real-world"[tiab] OR "effectiveness"[tiab] OR "barriers"[tiab] OR "facilitators"[tiab] OR "coach"[tiab] OR "coaching"[tiab] OR "training"[tiab] OR "education"[tiab] OR "program delivery"[tiab] OR "program implementation"[tiab] OR "feasibility"[tiab] OR "sustainability"[tiab] OR "dissemination"[tiab] OR "uptake"[tiab] OR "utilization"[tiab] OR "utilisation"[tiab]) AND ("Female"[MeSH Terms] OR "female athlete"[tiab] OR "women"[tiab] OR "girls"[tiab] OR "female"[tiab]) AND ("randomized controlled trial"[pt] OR "controlled clinical trial"[pt] OR "randomized"[tiab] OR "randomised"[tiab] OR "trial"[tiab] OR "crossover"[tiab] OR "cross-over"[tiab] OR "clinical trial"[tiab] OR "RCT"[tiab]) AND ("2014/01/01"[PDAT] : "2025/05/31"[PDAT]) AND "english"[lang] | 26 |
| Scopus | TITLE-ABS-KEY ( ( ( "anterior cruciate ligament" OR "ACL" OR "knee injur*" ) AND ( "injur* prevent*" OR "prevent*" OR "prevention" ) ) AND ( "neuromuscular training" OR "neuromuscular exercise*" OR "neuro-muscular" OR "neuro muscular" OR "proprioceptiv*" OR "balance training" OR "plyometric*" OR "strength training" OR "warm-up" OR "warmup" OR "warm up" OR "FIFA 11+" OR "FIFA11+" OR "PEP program*" OR "KLIP" OR "Sportsmetrics" OR "prevent* program*" OR "injur* prevent* program*" OR "exercise therapy" OR "training program*" ) AND ( "implement*" OR "complian*" OR "adheren*" OR "adoption" OR "real world" OR "real-world" OR "effectiveness" OR "barrier*" OR "facilitat*" OR "coach*" OR "coaching" OR "feasib*" OR "sustainab*" OR "disseminat*" OR "uptake" OR "utilizat*" OR "utilisation" OR "program delivery" OR "program implementation" OR "maintenance" OR "adoption rate*" ) AND ( "female*" OR "women" OR "girl*" OR "female athlete*" ) AND ( "athlete*" OR "sport*" OR "player*" OR "team sport*" OR "soccer" OR "football" OR "basketball" OR "handball" OR "volleyball" OR "netball" OR "lacrosse" OR "field hockey" ) AND ( "randomized" OR "randomised" OR "trial" OR "RCT" OR "controlled trial" OR "clinical trial" OR "crossover" OR "cross-over" OR "controlled study" ) ) AND PUBYEAR > 2013 AND PUBYEAR < 2025 AND ( LIMIT-TO ( SRCTYPE , "j" ) ) AND ( LIMIT-TO ( DOCTYPE , "ar" ) ) AND ( LIMIT-TO ( LANGUAGE , "English" ) ) AND ( LIMIT-TO ( EXACTKEYWORD , "Female" ) OR LIMIT-TO ( EXACTKEYWORD , "Human" ) ) AND ( LIMIT-TO ( PUBSTAGE , "final" ) ) | 59 |
| SportDiscus | ((TI ("anterior cruciate ligament" OR "ACL" OR "knee injur*") OR AB ("anterior cruciate ligament" OR "ACL" OR "knee injur*")) AND (TI ("injur* prevent*" OR "prevent*" OR "injury prevention") OR AB ("injur* prevent*" OR "prevent*" OR "injury prevention"))) AND ((TI ("neuromuscular training" OR "neuromuscular exercise*" OR "proprioceptiv* training" OR "balance training" OR "plyometric* training" OR "strength training" OR "warm-up program*" OR "FIFA 11+" OR "PEP program*" OR "KLIP program*" OR "Sportsmetrics" OR "prevent* program*") OR AB ("neuromuscular training" OR "neuromuscular exercise*" OR "proprioceptiv* training" OR "balance training" OR "plyometric* training" OR "strength training" OR "warm-up program*" OR "FIFA 11+" OR "PEP program*" OR "KLIP program*" OR "Sportsmetrics" OR "prevent* program*"))) AND ((TI ("implement*" OR "complian*" OR "adheren*" OR "real world" OR "real-world" OR "effectiveness" OR "barrier*" OR "facilitat*" OR "coach*" OR "feasib*" OR "sustainab*" OR "program delivery") OR AB ("implement*" OR "complian*" OR "adheren*" OR "real world" OR "real-world" OR "effectiveness" OR "barrier*" OR "facilitat*" OR "coach*" OR "feasib*" OR "sustainab*" OR "program delivery"))) AND ((TI ("female*" OR "women" OR "girl*" OR "female athlete*") OR AB ("female*" OR "women" OR "girl*" OR "female athlete*"))) AND ((TI ("athlete*" OR "sport*" OR "player*" OR "soccer" OR "football" OR "basketball" OR "handball" OR "volleyball") OR AB ("athlete*" OR "sport*" OR "player*" OR "soccer" OR "football" OR "basketball" OR "handball" OR "volleyball"))) AND ((TI ("randomized" OR "randomised" OR "RCT" OR "controlled trial" OR "clinical trial" OR "trial") OR AB ("randomized" OR "randomised" OR "RCT" OR "controlled trial" OR "clinical trial" OR "trial")))  Limited to: Full text, Peer reviewed; Language: English; Source Type; Academic Journal; | 10 |
| Web Of Science | TS=(("anterior cruciate ligament" OR "ACL" OR "knee injur*") AND ("injur* prevent*" OR "prevent*" OR "prevention")) AND TS=("neuromuscular training" OR "neuromuscular exercise*" OR "neuro-muscular" OR "neuro muscular" OR "proprioceptiv*" OR "balance training" OR "stability training" OR "plyometric*" OR "strength training" OR "resistance training" OR "warm-up" OR "warmup" OR "warm up" OR "FIFA 11+" OR "FIFA11+" OR "PEP program*" OR "KLIP" OR "Sportsmetrics" OR "prevent* program*" OR "injur* prevent* program*" OR "exercise therapy" OR "training program*" OR "intervention program*") AND TS=("implement*" OR "complian*" OR "adheren*" OR "adoption" OR "real world" OR "real-world" OR "effectiveness" OR "barrier*" OR "facilitat*" OR "coach*" OR "coaching" OR "feasib*" OR "sustainab*" OR "disseminat*" OR "uptake" OR "utilizat*" OR "utilisation" OR "program delivery" OR "program implementation" OR "translat*" OR "scale-up" OR "scaling" OR "maintenance" OR "fidelity") AND TS=("female*" OR "women" OR "girl*" OR "female athlete*" OR "women athlete*" OR "girls sport*") AND TS=("athlete*" OR "sport*" OR "player*" OR "team sport*" OR "soccer" OR "football" OR "basketball" OR "handball" OR "volleyball" OR "netball" OR "lacrosse" OR "field hockey" OR "rugby") AND TS=("randomized" OR "randomised" OR "randomized controlled trial*" OR "RCT" OR "controlled trial*" OR "clinical trial*" OR "crossover" OR "cross-over" OR "cross over" OR "controlled clinical trial*" OR "intervention study" OR "intervention studies") and Preprint Citation Index (Exclude – Database) and 2025 or 2024 or 2023 or 2022 or 2021 or 2020 or 2019 or 2018 or 2017 or 2016 or 2015 or 2014 (Publication Years) and Article or Clinical Trial (Document Types) and Web of Science Core Collection (Database) and Humans or Female (MeSH Headings) and English (Languages) and Review Article (Exclude – Document Types) | 51 |
